# Controlling wind turbine tower vibration under external force by applying control systems combination

**DOI:** 10.1038/s41598-024-68237-6

**Published:** 2024-08-10

**Authors:** Y. A. Amer, A. T. EL-Sayed, M. M. Agwa

**Affiliations:** 1 Department of Mathematics, Faculty of Science, Zagazig University, Zagazig, Egypt; 2 Department of Basic Science, Modern Academy for Engineering and Technology, Cairo, Egypt; 3 Department of Basic Science, Zagazig Higher Institute of Engineering & Tech, Zagazig, Egypt

**Keywords:** Wind turbine tower, Averaging technique, Cubic negative velocity control, Linear negative acceleration controller, Approximate solution, Applied mathematics, Software, Mechanical engineering

## Abstract

The global focus has recently shifted away from fuel-based power sources, and one of the most important projects for energy production is wind energy. To maintain low costs, the current research examines the problem of vibrations affecting wind turbine towers’ performance (WTTs). In particular, the tower, resulting from excessive vibrations, can negatively affect a structure’s power output and service life, as it can cause fatigue. Therefore, we conducted numerical tests on various types of controlled systems. Our tests revealed that combining a new technique cubic negative velocity control (CNVC) and linear negative acceleration control (LNAC) was the most effective and cost-efficient option for vibration damping. This solution was derived by using an approximation method for the averaging technique. The external force is an important component of a nonlinear dynamic system and can be characterized by two-degree-of-freedom (2-DOF) differential coupled equations. After implementing the control measures, we conducted a numerical analysis of the vibration values before and after the operation. Stability is studied numerically. The numerical and approximate solutions were confirmed through the frequency response equation and time history with fourth-order Runge–Kutta (RK-4). Finally, we investigated the effect of parameters and compared our results with those of previously published studies.

## Introduction

Vertical turbines, that harness wind energy to generate power are typically called air turbines or modern air mills. These devices create turbulence through a series of feathers, or streamlines, that are arranged vertically around a rotating axis or structure. Small wind turbines have a wide range of applications, including remote monitoring, off-grid housing, and powering telecom towers and rural clinics. Despite their usefulness, air turbines face a challenge in that larger turbines tend to produce more structural vibrations, which can shorten the lifespan of components such as blades, main shafts, bearings, generators, and gearboxes and may even lead to failure or destruction.

The tower is one of the necessary parts of a wind turbine because it is a vertical support system for the entire turbine assembly. It is crucial for regulating vibrations and offering stability, structural integrity, and support to different components of wind turbines. This explains the importance of controlling vibrations for the tower (principal structural elements, vibration control, dynamic response, protection of components, and safety).

Tong et al.^1^ discussed how to use the hydraulic reservoir of a floating barge hydrostatic wind turbine (HWT) as a bidirectional tuned liquid column damper (BTLCD) by shaping the reservoir into the shape of an annular rectangular. This effectively suppresses the pitch and roll motions of the barge. This lowers the tower load and improves power quality. Zhang et al.^2^ examined the vibration reduction potential of an under-actuated floating wind turbine through robust control and dynamic modeling. To reduce the loads of the under actuated floating wind turbine, a novel hierarchical sliding mode control system based on disturbance observers was created. The states of this type of under actuated nonlinear system are controlled by a hierarchical sliding mode controller. Verma et al.^3^ examined how a passive (TMD) affected the response-based limiting sea states as well as the impact velocities that appeared between the blade root and hub during the mating phase. Time domain multi-body simulations of an installation system have been carried out, and the efficiency of TMD for limiting the impact velocity has been measured.

Long et al.^4^ concentrated on examining the recurring loadings brought on by the rotor rotation when the tower surfers were in action. It was suggested that an active tuned mass damper (ATMD) with a virtual TMD algorithm be used to reduce the tower’s along-wind vibration when it is parked and in operation. Ultimately, the findings show that with varying wind speeds, a tuned mass damper (TMD) system with set parameters becomes out of tune and may become less effective. Nogueira et al.^5^ introduced a technique for structural management of wind turbines utilizing floating offshore wind turbines (FOWT) by employing active control. A proportional-derivative controller and a tuned mass damper (TMD) make up the suggested control system. Subsequent analysis of the two systems reveals that the suggested control method minimizes oscillations in generator speed and output power as well as better mitigates platform pitch angle oscillations.

Eissa and Sayed^6^ employed a transversally tuned absorber and linked it to a spring-pendulum system (3DOF) that was externally activated and under harmonic excitation. They used negative displacement feedback control to change the system’s linear frequency and shift it away from the resonant one. They also used negative velocity feedback, or its square or cubic value, to improve the spring-pendulum’s behavior at the primary resonance. Ultimately, the system’s ideal operating conditions were determined through the use of both passive and active control techniques, which will be applied in the system’s design. Jun et al.^7^ studied Non-linear analysis, design, and vibration isolation for a bilinear system with time-delayed cubic velocity feedback and applied the perturbation method of multiple scales to find approximate solutions.

Sayed et al.^8^ and Sayed and Bauomy^9^ explained the importance of controlling vibration in these systems to reduce or eliminate the risk of damage or destruction because vibration and dynamical chaos can be undesirable phenomena. For example, the dynamical response of mechanical and civil structures subject to high amplitude vibration can frequently cause disturbance, discomfort, damage, danger, and structural destruction. Under external and parametric excitation forces, the nonlinear dynamics of a (3DOF) vibration system with quadratic and cubic nonlinearities are examined. Negma and Maalawi^10^ explained several optimization models for the standard wind turbine tower structural design. They also created and evaluated five optimization solutions. The last one, which lowers vibration levels by directly maximizing the natural frequencies of the system, functions admirably and produces fantastic outcomes for both the tower by itself and the tower/rotor model together.

Jalbi et al.^11^ finite element approach has been used to evaluate analytical solutions that have been developed to estimate the Eigen frequencies of offshore wind turbines supported by jackets. They discovered that the aspect ratio and the ratio of superstructure stiffness control a jacket’s rocking frequency. Finally, they predicted that the findings would have a positive influence on the selection of jacket foundations. Sarkar and Fitzgerald^12^ discuss using a passively tuned mass-damper-inverter (TMDI) to dampen vibration in offshore wind turbine towers of the spar type. They detailed their plan to construct a passive TMDI for an offshore wind turbine tower. The outcomes have demonstrated the TMDI’s significant benefits over the traditional TMD, resulting in remarkable response reductions with smaller, more tuned mass strokes.

Andrade et al.^13^ examined a lower-limb exoskeleton with six degrees of freedom (6-DOF)to develop a framework model based on the double-pendulum method combined with the mechanical impedance of the actuators. The findings indicate that, firstly, the independent impact of these factors on the necessary driving torques for the system is provided. The modifications in friction, link length, and inertia had a significant impact on the oscillation frequency for the minimum joint torque. Second, depending on the oscillation frequency, a heavier exoskeleton with a low-ratio transmission needed less torque and mechanical power than a lighter one with a bigger reduction ratio due to the combined effects of the actuator’s mass, inertia, and friction. Amer et al.^14^ examined the movement of (3-DOF) double nonlinear damped spring pendulums. Lagrange’s equations are used to generate the equation of motion, which is then asymptotically solved using the many-scales approach up to the third approximation. The obtained data, response resonance curves, and stability regions are explored and graphically depicted to examine the favorable effect of the parameters on the system under investigation’s dynamic behavior.

Awada et al.^15^ provided an extensive research assessment of the various mitigation strategies as well as a detailed presentation of the issues related to wind turbine vibration. Furthermore, they investigated the benefits, limitations, and difficulties associated with the current vibration control technologies for wind turbines. Based on their operation to reduce vibrations and the physical principles they employ; these systems fall into six primary groups. They completed a multi-criteria study of several systems that were at various stages of development. Yang et al.^16^ investigated a 10 MW offshore wind turbine’s dynamic reactions. The results showed that the condition of the tendons has a significant impact on the platform’s dynamic behavior. Furthermore, there was a 165% increase in the magnitude of the peak tension in the tendon that was next to the broken tendon. Tendon breakages have a little impact on the platform’s collective-pitch mode and wave excitation, which are the primary causes of the surge and pitch variations. Furthermore, distinct fractal characteristics from the multiracial spectra of the platform accelerations under various tendon failure scenarios demonstrate the necessity of tendon failure detection and diagnosis for the development of a structural health monitoring system for FOWTs.

Amer et al.^17,18^ evaluated the design of an auto-parametric system with (3-DOF) and its new vibrating dynamical motion. The perturbation technique of various scales was then employed to offer the solutions to these equations up to a higher level of approximation. Finally, they anticipated that these findings would be useful for specialized studies in the domains of mechanics and space engineering, as well as studies about the vibration of swaying structures and the attenuation of rotor dynamics vibration. Wang et al.^19^ constituted the Lagrange theory was used to derive the IMD vibration isolator’s dynamic equation. The harmonic balancing and pseudo-arc-length methods were used to determine the dynamic response under base harmonic excitation, and the stability of the dynamic response is taken into account. To achieve improved shock and isolation performance. As a result, the suggested IMD vibration isolator’s design demonstrates the benefits of using an inverter and offers superior isolation and shock performance in a variety of orientations. Kandil et al.^20^ researched the cross-sectional defects, both vertically and horizontally, of a revolving beam with thin walls, and analysis was conducted across several time scales. Then, to submit the beam to a simultaneous resonance of 1:1:1, the rotation speed of the beam was changed to be close to both of the natural frequencies of the defections.

Zhang et al.^21^ used pseudo-arc-length, harmonic balancing, and numerical approaches, the dynamic response of the system under pavement harmonic and random excitations was determined. Based on the data, it was found that employing NESIs lessens rear dynamic tire loads, resonance peaks, and RMS values for various performance measures. Lei et al.^22,23^ studied the control performance generated by a flexible cable deformation effect using a unique prestressed tuned mass damper (PS-TMD) because larger megawatt wind turbines are significantly taller and can experience excessive vibration under external dynamic stimulation. They discovered that the nonlinear PS-TMD performs better at mitigating vibrations than the linear PS-TMD and that PS-TMD can reduce vibration amplitude more than PS-TMD. Amer et al.^24^ used the multiple-scale method to approximate the solution of the vibrating system and then used the frequency response equation to investigate the stability of the controlled system. They presented a mix of active controller negative velocity and acceleration feedback to eliminate the vibration of the nonlinear oscillations of a continuously rotating shaft in one of the worst simultaneous resonance cases. The approximate solution derived from the multiple scales and the numerical solution shows good agreement.

Li et al.^25^ examined the mechanism of energy transfer between the (2-DOF) of rotation and vibration in a cantilever beam system. The two nonlinear peaks on the rotation DOF are highly favorable for energy harvesting in the broad frequency domain when there is internal resonance between 2-DOFs, according to the analytical equation simulation. Additionally, they investigated every parameter of the energy harvesting system’s amplitude response and resonance frequency. Amer et al.^26^ reviewed the vibrational behavior of a 2-DOF spring pendulum on a plane, where the pivot point is restricted to travel along a Lissajous curve. To produce unique approximate solutions, they employed the standard approach of multiple scales (AMS). These were compared with numerical solutions acquired by the application of the algorithm (RK-4) to get analytic solutions. Moatimid et al.^27^ utilized nonlinear control theory to regulate the pendulum’s motion. They applied a numerical approach based on the (RK-4) method to provide support for an earlier answer. To mathematically model a 2DOF system that represents the IP with the PPF, the researchers presented a second-order multiple time scale perturbation methodology. Additionally, the stability of the IP was improved by the magnetic field. Kandil and Hamed^28^ used controller is the integral resonant controller (IRC), which is a first-order oscillator coupled to the car via a linear variable differential transformer (LVDT) and a servo-controlled linear actuator (SCLA). The multiple-scale perturbation method is used to obtain an approximate solution, and stability analyses are carried out.

Ultimately, the role of the wind turbine tower in managing vibrations is crucial to the reliable and efficient operation of wind turbines. Through their precise design, construction, and maintenance, wind turbine towers enhance the efficiency, durability, and safety of wind energy systems. Thus, the primary goal is to establish a control system for windmills that can effectively manage vibrations, which is a critical aspect of energy production, and to offer various types of control systems while comparing numerical and mathematical solutions to verify the outcomes.

## Mechanical model

The horizontal displacement $x_{1} \left( {z, \, t} \right)$ of the tower section at height z can be represented using the generalized displacement concept as follows: $x_{1} \left( {z, \, t} \right) \, = \, \varphi_{I} \left( z \right) \, x_{1} \left( t \right)$ where $t$ is the time, $x_{1} \left( t \right)$ is the generalized displacement of the tower top, and φ_I_ (z) indicates the value of the shape function for the first vibration mode. The generalized displacement of the IP-TMD is $x_{2} \left( t \right)$. $H$ is the tower height, and $h$ is the separation between the steel WTT tower top and the uppermost inner platform. The distribution mass coefficient of the tower section at height Z is indicated by the symbol $M\left( z \right)$. The relative displacement between the IP-TMD and the tower wall is shown by $Q$. From Newton’s movement equations, the motion equations have been used to deduce the dynamic system, as shown in Ref.^29^.

**Figure 1:**
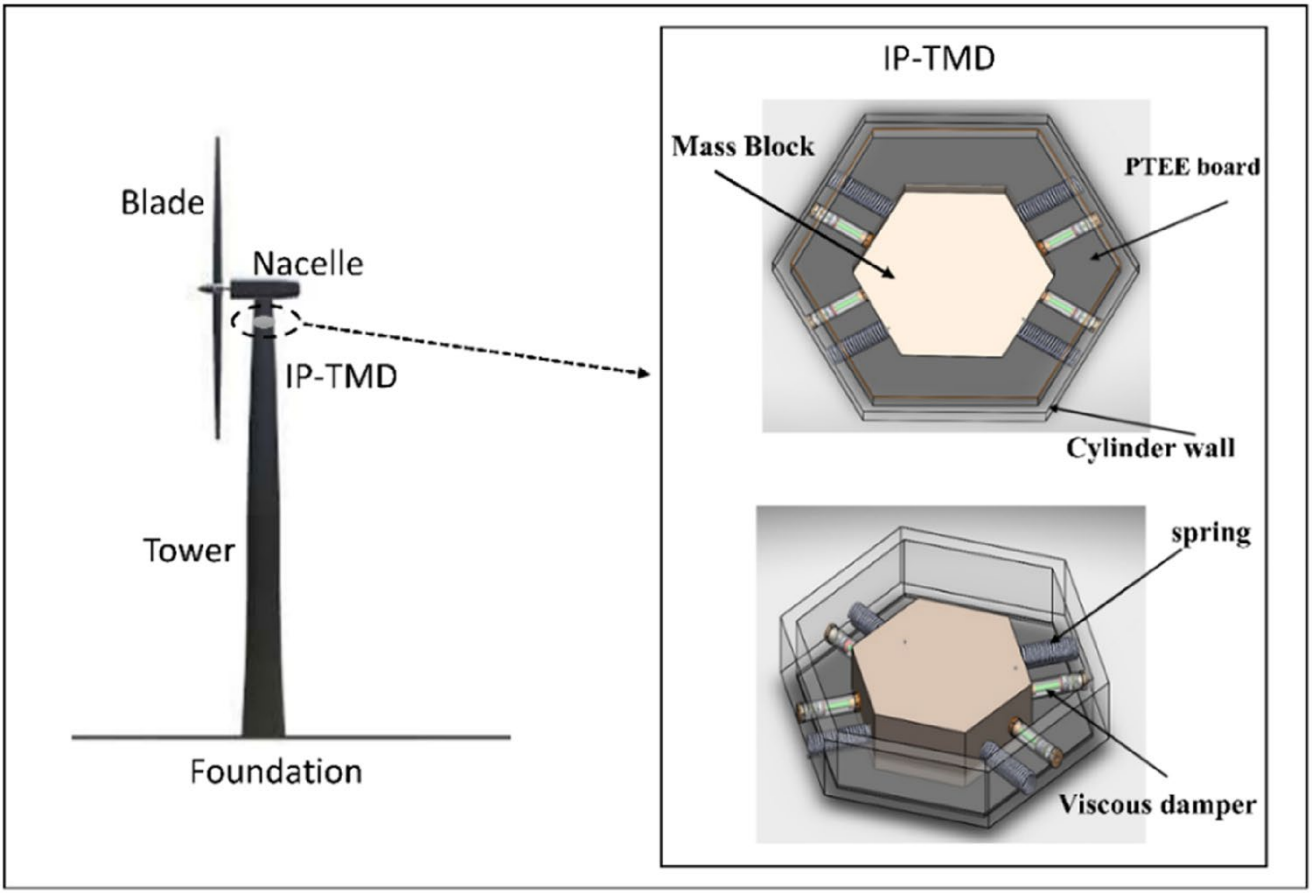
Schematic of steel WTT with the IP-TMD device.

Figure 1 shows the structures of (TP) and (TMD). Generally, dampers are positioned carefully throughout a building’s structure to manage floor vibrations and building displacement and to reduce the impact of significant seismic occurrences. The dampers absorb and release the energy produced by the floor vibration and building displacement. In a steel WTT, the figure study (TP-TMD) is performed in a certain location to regulate the tilt and vibration of the tower. A device installed in structures to reduce mechanical vibrations is called a (TMD), which is sometimes referred to as a seismic damper or harmonic absorber. A mass, spring, and damper are components of a (TMD), which is fastened to a structure to reduce its dynamic response. Wind turbine tower vibrations can be significantly reduced by passive solutions such as (TMDs). This is how they are connected.

- Control of vibrations.
- Damping of resonance.
- Improved structural efficiency.
- Flexibility in response to site conditions.
- Cost-effectiveness.

Generally speaking, improving the structural performance, lowering vibrations, and increasing the overall dependability and efficiency of wind energy systems may all be achieved by including passive schemes such as TMDs in wind turbine tower designs.

**Figure 2:**
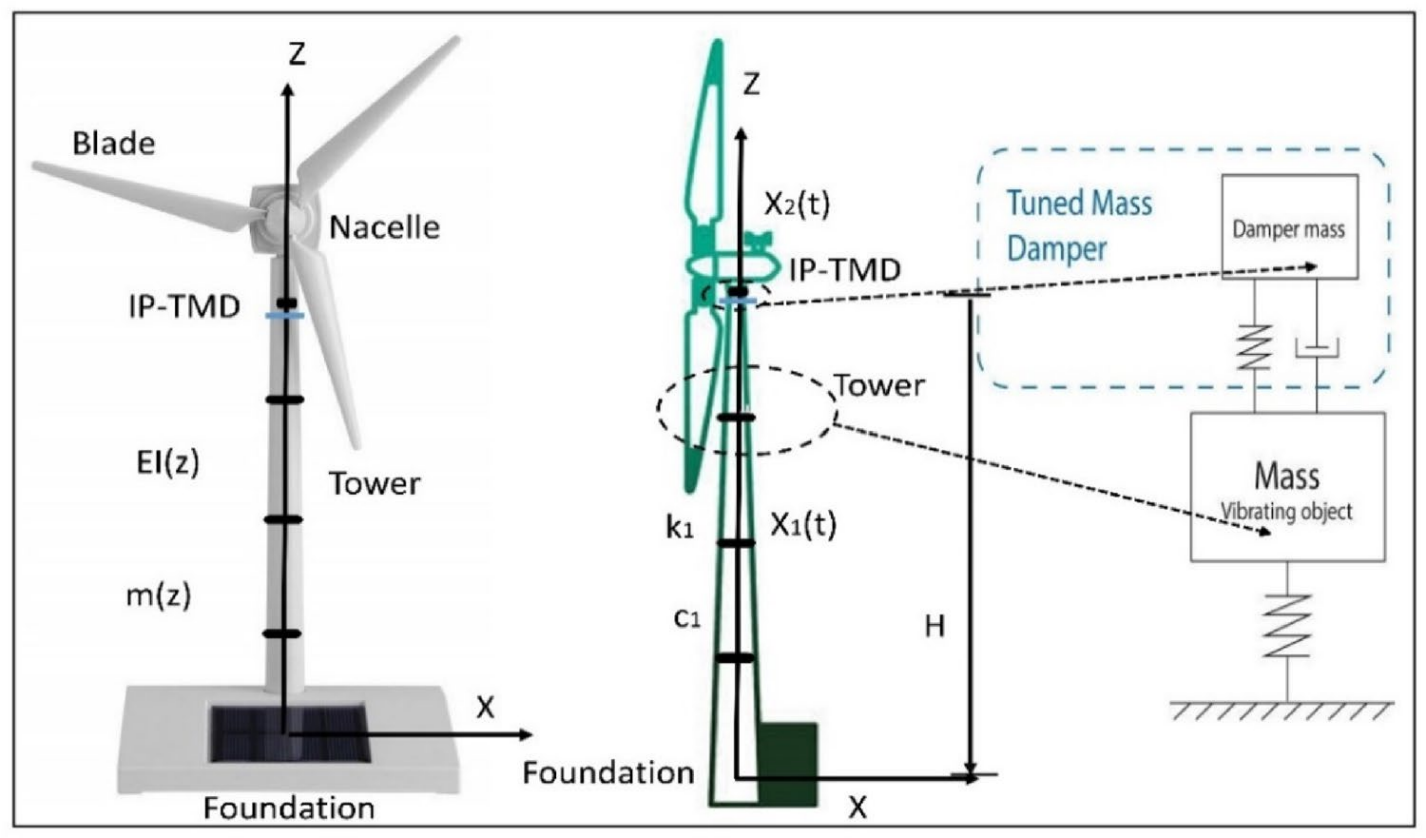
Mechanical model of WTT controlled by IP-TMD.

Owing to its significance, the vertical structure that holds up the other components of the wind turbine was examined. As shown in Fig. 2, a genuine steel wind turbine’s basic supporting structure is made up of a tower, foundation, nacelle, and three blades. Conical pieces with thin walls that varied in diameter and wall thickness were used for assembly. The tower was composed of nine pieces that varied in diameter, wall thickness, and degree of inclination.

## Equation of motion

**Figure 3:**
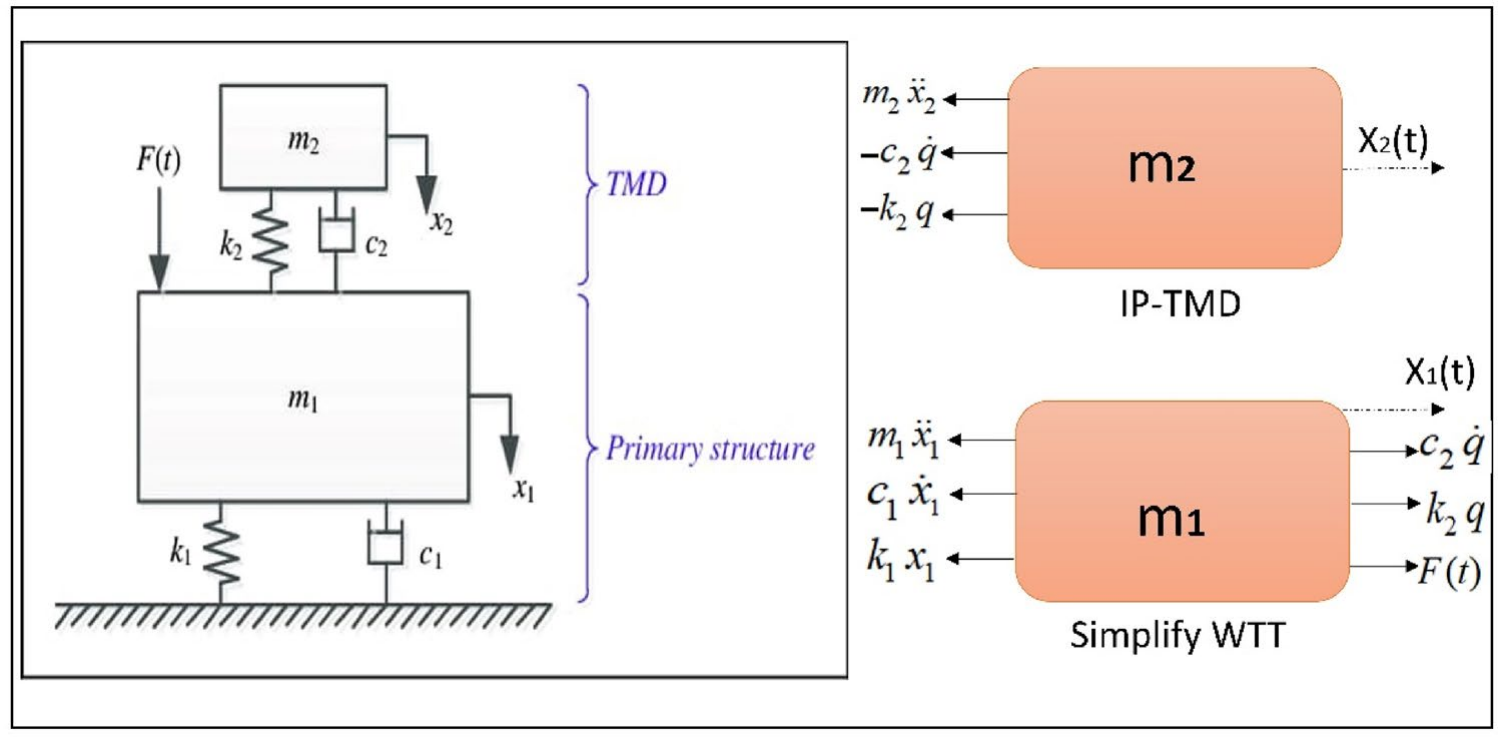
Mechanical diagram for steel WTT with the IP-TMD device.

The equation of motion can be obtained from free-body diagrams based on Newton’s second law of motion, $F = \, ma$, as shown in Fig. 3 The strength of the system was analyzed to find a free body diagram, as explained, and the production of motion equations. We explain the mathematical model of dynamical system consisting (2-DOF) differential coupled equations and the system’s equation, as shown in Fig. 3.

Where $$q = \left( {x_{2} - x_{1} } \right)$$.

The equation of motion can therefore be expressed as:

1a $$m_{1} \ddot{x}_{1} = - c_{1} \dot{x}_{1} - k_{1} x_{1} + c_{2} [\dot{x}_{2} - \dot{x}_{1} ] + k_{2} [x_{2} - x_{1} ] + f_{eff} (t),$$

1b $$m_{2} \ddot{x}_{2} = - c_{2} [\dot{x}_{2} - \dot{x}_{1} ] - k_{2} [x_{2} - x_{1} ],$$

2a $$m_{1} \ddot{x}_{1} + c_{1} \dot{x}_{1} + k_{1} x_{1} + \varphi_{\rm I} c_{2} [\varphi_{\rm I} \dot{x}_{1} - \dot{x}_{2} ] + \varphi_{\rm I} k_{2} [\varphi_{\rm I} x_{1} - x_{2} ] = f_{eff} (t),$$

2b $$m_{2} \ddot{x}_{2} + c_{2} [\dot{x}_{2} - \varphi_{\rm I} \dot{x}_{1} ] - k_{2} [\varphi_{\rm I} x_{1} - x_{2} ] = 0.$$

where $$x_{1} (z,t) = \varphi_{I} (z)\,x_{1} (t)$$

3a $$\ddot{x}_{1} + \omega_{1}^{2} x_{1} + \alpha_{1} \dot{x}_{1} + \gamma_{1\,} [\varphi_{\rm I} \dot{x}_{1} - \dot{x}_{2} ] + \gamma_{2} [\varphi_{\rm I} x_{1} - x_{2} ] = f\,\sin (\omega t),$$

3b $$\ddot{x}_{2} + \omega_{2}^{2} x_{2} + \alpha_{2} [\dot{x}_{2} - \varphi_{\rm I} \dot{x}_{1} ] - \gamma_{3} [x_{1} ] = 0,$$

where $$\omega_{1}^{2} = \frac{{k_{1} }}{{m_{1} }},\,\alpha_{1} = \,\frac{{c_{1} }}{{m_{1} }},\,\,\gamma_{1\,} = \frac{{\varphi_{\rm I} c_{2} }}{{m_{1} }},\gamma_{2} = \frac{{\varphi_{\rm I} k_{2} }}{{m_{1} }},\,f = \frac{1}{{m_{1} }}p_{o}$$, $$\omega_{2}^{2} = \frac{{k_{2} }}{{m_{2} }},\,\,\alpha_{2} = \,\frac{{c_{2} }}{{m_{2} }},\,\,\gamma_{3\,} = \frac{{\varphi_{\rm I} k_{2} }}{{m_{1} }}.$$

## Equations of motion with types different of control

We modified the equation of (WTTs) system by adding five different types of the controllers

4a $$\ddot{x}_{1} + \omega_{1}^{2} x_{1} + \alpha_{1} \dot{x}_{1} + \gamma_{1} [\varphi_{\rm I} \dot{x}_{1} - \dot{x}_{2} ] + \gamma_{2} [\varphi_{\rm I} x_{1} - x_{2} ] = f\sin (\omega t) + F_{A} (t),$$

4b $$\ddot{x}_{2} + \omega_{2}^{2} x_{2} + \alpha_{2} [\dot{x}_{2} - \varphi_{\rm I} \dot{x}_{1} ] - \gamma_{3} [x_{1} ] = F_{B} (t),$$

where $F_{A} \left( t \right)$ and $F_{B} \left( t \right)$ are the control inputs, it can express the different types of controls to decrease the vibration as follows:

First type: cubic linear negative velocity (CNVC)

5a $$F_{A} (t) = G_{1} \dot{x}_{1}^{3} ,$$

5b $$F_{B} (t) = G_{2} \dot{x}_{2}^{3} .$$

Second type: linear negative velocity (LNVC)

6a $$F_{A} (t) = G_{1} \dot{x}_{1} ,$$

6b $$F_{B} (t) = G_{2} \dot{x}_{2} .$$

Third type: proportional derivative (PD)

7a $$F_{A} (t) = - G_{1} x_{1} - G_{2} \dot{x}_{1} ,$$

7b $$F_{B} (t) = - G_{3} x_{2} - G_{4} \dot{x}_{2} ,$$

where proportional gain $G_{1} ,G_{3}$ and $G_{2} ,G_{4}$ derivative gain.

Fourth type: CNVC and negative acceleration control (LNAC)

8a $$F_{A} (t) = G_{1} \dot{x}_{1}^{3} - G_{2} \ddot{x}_{1} ,$$

8b $$F_{B} (t) = G_{3} \dot{x}_{2}^{3} - G_{4} \ddot{x}_{2} .$$

Fifth type: positive position feedback (PPF)

9a $$\ddot{u} + \omega_{1}^{2} \dot{u} + \alpha_{3} u = G_{u} \eta_{1} ,$$

9b $$\ddot{v} + \omega_{4}^{2} \dot{v} + \alpha_{4} v = G_{v} \eta_{2} ,$$

$$F_{A} (t) = G_{1} u,\;F_{B} (t) = G_{2} v\;{\text{and}}\;G_{u} \eta_{1} = G_{3} x_{1} ,\;G_{v} \eta_{2} = G_{4} x_{2} .$$

Sixth type: negative derivative feedback (NDF)

10a $$\ddot{u} + \omega_{1}^{2} \dot{u} + \alpha_{3} u = G_{u} \eta_{1} ,$$

10b $$\ddot{v} + \omega_{4}^{2} \dot{v} + \alpha_{4} v = G_{v} \eta_{2} ,$$

$$F_{A} (t) = G_{1} \dot{u},\;F_{B} (t) = G_{2} \dot{v}\;{\text{and}}\;G_{u} \eta_{1} = - G_{3} \dot{x}_{1} ,\;G_{v} \eta_{2} = - G_{4} \dot{x}_{2} ,$$

where $G_{1} ,G_{2}$ gain of control and $G_{3} ,G_{4}$ feedback gain.

### Equations of motion with (cubic NVC and negative acceleration)

After making a numerical comparison with the addition of different types of controllers in our system as presented in (Fig. 15, Table 1) we found that the better control system is a mix between (CNVC) and (LNAC). Then the equations of the motion are deduced as the ones that are the same:

11 $$\ddot{x}_{1} + \omega_{1}^{2} x_{1} + \alpha_{1} \dot{x}_{1} + \gamma_{1} [\varphi_{\rm I} \dot{x}_{1} - \dot{x}_{2} ] + \gamma_{2} [\varphi_{\rm I} x_{1} - x_{2} ] = f\sin (\omega t) - G_{1} \dot{x}_{1}^{3} - G_{2} \ddot{x}_{1} ,$$

12 $$\ddot{x}_{2} + \omega_{2}^{2} x_{2} + \alpha_{2} [\dot{x}_{2} - \varphi_{\rm I} \dot{x}_{1} ] - \gamma_{3} [x_{1} ] = - G_{3} \dot{x}_{2}^{3} - G_{4} \ddot{x}_{2} ,$$

where $\left( {\alpha_{1} ,\alpha_{2} } \right)$ result of dividing the damping coefficient of WTT by mass M_1_ and the damping coefficient of IP-TMD by mass M_2_, and $\left( {\gamma_{1} ,\gamma_{2} ,\gamma_{3} } \right)$ geometrically linear, inertia linearity coefficient of steel WTT and (TP-TMD).

**Table 1:** The values of absolute error and percentage error.

Type of control	Parameter of control	Effective of control $E_{a} = \frac{{V_{A1} }}{{V_{A2} }}$		Percentage of E_a_ $E_{a} = \left| {\frac{{V_{A1} }}{{V_{A2} }}} \right| \times 100\%$	
		Amplitude of $x_{1}$	Amplitude of $x_{2}$	Amplitude of $x_{1}$ (%)	Amplitude of $x_{2}$ (%)
NDF	$G_{1} = G_{2} = 1.5$	6.3	35.6	630	3560
PPF	$G_{1} = G_{2} = 1.5$	5.72	31.64	572	3164
LNVC	$G_{1} = G_{2} = 1.2$	1.512	1.726	151.2	172.6
Cubic NVC	$G_{1} = G_{2} = 0.9$	2.73	3.2	273	320
PD	$$\begin{gathered} G_{1} = G_{2} = 1.5 \hfill \\ G_{3} = G_{4} = 0.9 \hfill \\ \end{gathered}$$	1.4	1.498	140	149.8
CNVC and LNAC	$$\begin{gathered} G_{1} = G_{3} = 0.05 \hfill \\ G_{2} = G_{4} = 9 \hfill \\ \end{gathered}$$	7.41	28.48	741	2848

## Mathematical analysis

The approximate solution of the first order for the controlled system can be obtained by applying Eqs. (11) and (12), which allow the averaging technique to solve analytically. This equation’s necessary solution is expressed as follows:

13 $$x_{1} = a_{1} \cos \left( {\omega_{1} t + \varphi_{1} } \right),$$

14 $$x_{2} = a_{2} \cos \left( {\omega_{2} t + \varphi_{2} } \right),$$

where $\omega_{n} ,\varphi_{n} ,a_{n}$ (n = 1, 2) are constant. Differentiating Equations that (13) and (14) concerning $t$ yields:

15 $$\dot{x}_{1} = - \omega_{1} a_{1} \sin \left( {\omega_{1} t + \varphi_{1} } \right),$$

16 $$\dot{x}_{2} = - \omega_{2} a_{2} \sin \left( {\omega_{2} t + \varphi_{2} } \right).$$

Let $\omega_{n} ,\varphi_{n}$ and $a_{n}$ are unknown functions of time $t$ in Eqs. (11) and (12). Then, differentiating Eqs. (14) and (15) concerning $t$, then comparing with Eqs. (16) and (17), we get:

17 $$\dot{\varphi }_{1} a_{1} \sin \left( {\omega_{1} t + \varphi_{1} } \right) - \dot{a}_{1} \cos \left( {\omega_{1} t + \varphi_{1} } \right) = 0,$$

18 $$\dot{\varphi }_{2} a_{2} \sin \left( {\omega_{2} t + \varphi_{2} } \right) - \dot{a}_{2} \cos \left( {\omega_{2} t + \varphi_{2} } \right) = 0.$$

Differentiating Eqs. (15) and (16) once concerning $t$, we get:

19 $$\ddot{x}_{1} = - \omega_{1} \dot{a}_{1} \sin \left( {\omega_{1} t + \varphi_{1} } \right) - \omega_{1}^{2} a_{1} \cos \left( {\omega_{1} t + \varphi_{1} } \right) - \omega_{1} a_{1} \dot{\varphi }_{1} \cos \left( {\omega_{1} t + \varphi_{1} } \right),$$

20 $$\ddot{x}_{2} = - \omega_{2} \dot{a}_{2} \sin \left( {\omega_{2} t + \varphi_{2} } \right) - \omega_{2}^{2} a_{2} \cos \left( {\omega_{2} t + \varphi_{2} } \right) - \omega_{2} a_{2} \dot{\varphi }_{2} \cos \left( {\omega_{2} t + \varphi_{2} } \right).$$

Sub from Eqs. (13)–(16), (19) and (20) in Eqs. (11) and (12), we obtained:

21 $$\begin{aligned} - & \left( {1 + G_{2} } \right)\omega_{1} \dot{a}_{1} \sin \left( {\omega_{1} t + \varphi_{1} } \right) - \left( {1 + G_{2} } \right)\omega_{1} a_{1} \dot{\varphi }_{1} \cos \left( {\omega_{1} t + \varphi_{1} } \right) \\ & = \alpha_{1} a_{1} \omega_{1} \sin \left( {\omega_{1} t + \varphi_{1} } \right) + \gamma_{1} \varphi_{\rm I} a_{1} \omega_{1} \sin \left( {\omega_{1} t + \varphi_{1} } \right) - \gamma_{1} a_{2} \omega_{2} sin\left( {\omega_{2} t + \varphi_{2} } \right) \\ & \quad - \gamma_{2} \varphi_{\rm I} a_{1} \cos \left( {\omega_{1} t + \varphi_{1} } \right) + \gamma_{2} a_{2} \cos \left( {\omega_{2} t + \varphi_{2} } \right) + f\sin (\omega t) + G_{1} a_{1}^{3} \omega_{1}^{3} \sin^{3} \left( {\omega_{1} t + \varphi_{1} } \right) \\ & \quad + G_{2} \omega_{1}^{2} a_{1} \cos \left( {\omega_{1} t + \varphi_{1} } \right), \\ \end{aligned}$$

22 $$\begin{aligned} - & \left( {1 + G_{4} } \right)\omega_{2} \dot{a}_{2} \sin \left( {\omega_{2} t + \varphi_{2} } \right) - \left( {1 + G_{4} } \right)\omega_{2} a_{2} \dot{\varphi }_{2} \cos \left( {\omega_{2} t + \varphi_{2} } \right) \\ & = \alpha_{2} a_{2} \omega_{2} sin\left( {\omega_{2} t + \varphi_{2} } \right) - \alpha_{2} \varphi_{\rm I} a_{1} \omega_{1} \sin \left( {\omega_{1} t + \varphi_{1} } \right) + \gamma_{3} a_{1} \cos \left( {\omega_{1} t + \varphi_{1} } \right) \\ & \quad + G_{3} a_{2}^{3} \omega_{2}^{3} sin^{3} \left( {\omega_{2} t + \varphi_{2} } \right) + G_{4} \omega_{2}^{2} a_{2} \cos \left( {\omega_{2} t + \varphi_{2} } \right). \\ \end{aligned}$$

By using the Trigonometric function and substituting from Eqs. (17) and (18) in Eqs. (21) and (22) and simplifying we obtained equations of $\dot{a}_{1} ,\dot{a}_{2} ,a_{1} \dot{\varphi }_{1} ,a_{2} \dot{\varphi }_{2}$.

Following a numerical analysis, every potential resonance case can be identified. The worst resonance situations were found using numerical checking, allowing for an analysis of the system’s stability. The chosen examples of resonance are:

23 $$\begin{gathered} \omega_{1} = \omega_{2} + \varepsilon \sigma_{1} \hfill \\ \omega_{1} = \omega + \varepsilon \sigma_{2} , \hfill \\ \end{gathered}$$

where $\sigma_{1} ,\sigma_{2}$ are the detuning parameters.

Using only the constant portions and slowly moving parts in Eqs. (17), (18) and (23) in Eqs. (21) and (22), we can introduce the equations as follows:

24 $$\begin{aligned} \dot{a}_{1} = - & \left( {\frac{{\alpha_{1} a_{1} }}{{2\left( {1 + G_{2} } \right)}} + \frac{{\gamma_{1} \varphi_{\rm I} a_{1} }}{{2\left( {1 + G_{2} } \right)}} + \frac{{3G_{1} a_{1}^{3} \omega_{1}^{2} }}{{8\left( {1 + G_{2} } \right)}}} \right) + \frac{{\gamma_{1} a_{2} \omega_{2} }}{{2\left( {1 + G_{2} } \right)\omega_{1} }}\cos \left( {\left( {\omega_{1} - \omega_{2} } \right)t + \left( {\varphi_{1} - \varphi_{2} } \right)} \right) \\ - & \frac{{\gamma_{2} a_{2} }}{{2\left( {1 + G_{2} } \right)\omega_{1} }}\sin \left( {\left( {\omega_{1} - \omega_{2} } \right)t + \left( {\varphi_{1} - \varphi_{2} } \right)} \right) - \frac{f}{{2\left( {1 + G_{2} } \right)\omega_{1} }}\cos \left( {\left( {\omega_{1} - \omega } \right)t + \left( {\varphi_{1} } \right)} \right), \\ \end{aligned}$$

25 $$\begin{aligned} a_{1} \dot{\varphi }_{1} = - & \frac{{\gamma_{1} a_{2} \omega_{2} }}{{2\left( {1 + G_{2} } \right)\omega_{1} }}\sin \left( {\left( {\omega_{1} - \omega_{2} } \right)t + \left( {\varphi_{1} - \varphi_{2} } \right)} \right) - \frac{{\gamma_{2} a_{2} }}{{2\left( {1 + G_{2} } \right)\omega_{1} }}\cos \left( {\left( {\omega_{1} - \omega_{2} } \right)t + \left( {\varphi_{1} - \varphi_{2} } \right)} \right) \\ + & \frac{{\gamma_{2} \varphi_{\rm I} a_{1} }}{{2\left( {1 + G_{2} } \right)\omega_{1} }} + \frac{f}{{2\left( {1 + G_{2} } \right)\omega_{1} }}\sin \left( {\left( {\omega_{1} - \omega } \right)t + \left( {\varphi_{1} } \right)} \right) - \frac{{G_{2} \omega_{1} a_{1} }}{{2\left( {1 + G_{2} } \right)}}, \\ \end{aligned}$$

26 $$\begin{aligned} \dot{a}_{2} = - & \frac{{\alpha_{2} a_{2} }}{{2\left( {1 + G_{4} } \right)}} + \frac{{\alpha_{2} \varphi_{\rm I} a_{1} \omega_{1} }}{{2\left( {1 + G_{4} } \right)\omega_{2} }}\cos \left( {\left( {\omega_{1} - \omega_{2} } \right)t + \left( {\varphi_{1} - \varphi_{2} } \right)} \right) - \frac{{3G_{3} a_{2}^{3} \omega_{2}^{2} }}{{8\left( {1 + G_{4} } \right)}} \\ + & \frac{{\gamma_{3} a_{1} }}{{2\left( {1 + G_{4} } \right)\omega_{2} }}\sin \left( {\left( {\omega_{1} - \omega_{2} } \right)t + \left( {\varphi_{1} - \varphi_{2} } \right)} \right), \\ \end{aligned}$$

27 $$\begin{aligned} a_{2} \dot{\varphi }_{2} = & \frac{{\alpha_{2} \varphi_{\rm I} a_{1} \omega_{1} }}{{2\left( {1 + G_{4} } \right)\omega_{2} }}\sin \left( {\left( {\omega_{1} - \omega_{2} } \right)t + \left( {\varphi_{1} - \varphi_{2} } \right)} \right) - \frac{{\gamma_{3} a_{1} }}{{2\left( {1 + G_{4} } \right)\omega_{2} }}\cos \left( {\left( {\omega_{1} - \omega_{2} } \right)t + \left( {\varphi_{1} - \varphi_{2} } \right)} \right) \\ & - \frac{{G_{4} \omega_{2} a_{2} }}{{2\left( {1 + G_{4} } \right)}}, \\ \end{aligned}$$

when $\omega_{1} - \omega_{2} = \varepsilon \sigma_{1} ,\,\,\,\omega_{1} - \omega = \varepsilon \sigma_{2}$ obtained that $\theta_{1} = \sigma_{1} t + \left( {\varphi_{1} - \varphi_{2} } \right),\,\,\theta_{2} = \sigma_{2} t + \left( {\varphi_{1} } \right)$. Presented the previous equations as the following:

28 $$\begin{aligned} \dot{a}_{1} = - & \left( {\frac{{\alpha_{1} a_{1} }}{{2\left( {1 + G_{2} } \right)}} + \frac{{\gamma_{1} \varphi_{\rm I} a_{1} }}{{2\left( {1 + G_{2} } \right)}} + \frac{{3G_{1} a_{1}^{3} \omega_{1}^{2} }}{{8\left( {1 + G_{2} } \right)}}} \right) + \frac{{\gamma_{1} a_{2} \omega_{2} }}{{2\left( {1 + G_{2} } \right)\omega_{1} }}\cos \left( {\theta_{1} } \right) - \frac{{\gamma_{2} a_{2} }}{{2\left( {1 + G_{2} } \right)\omega_{1} }}\sin \left( {\theta_{1} } \right) \\ - & \frac{f}{{2\left( {1 + G_{2} } \right)\omega_{1} }}\cos \left( {\theta_{2} } \right), \\ \end{aligned}$$

29 $$\begin{aligned} a_{1} \dot{\varphi }_{1} = - & \frac{{\gamma_{1} a_{2} \omega_{2} }}{{2\left( {1 + G_{2} } \right)\omega_{1} }}\sin \left( {\theta_{1} } \right) - \frac{{\gamma_{2} a_{2} }}{{2\left( {1 + G_{2} } \right)\omega_{1} }}\cos \left( {\theta_{1} } \right) - \frac{{G_{2} \omega_{1} a_{1} }}{{2\left( {1 + G_{2} } \right)}} + \frac{{\gamma_{2} \varphi_{\rm I} a_{1} }}{{2\left( {1 + G_{2} } \right)\omega_{1} }} \\ + & \frac{f}{{2\left( {1 + G_{2} } \right)\omega_{1} }}\sin \left( {\theta_{2} } \right), \\ \end{aligned}$$

30 $$\dot{a}_{2} = - \frac{{\alpha_{2} a_{2} }}{{2\left( {1 + G_{4} } \right)}} - \frac{{3G_{3} a_{2}^{3} \omega_{2}^{2} }}{{8\left( {1 + G_{4} } \right)}} + \frac{{\alpha_{2} \varphi_{\rm I} a_{1} \omega_{1} }}{{2\left( {1 + G_{4} } \right)\omega_{2} }}\cos \left( {\theta_{1} } \right) + \frac{{\gamma_{3} a_{1} }}{{2\left( {1 + G_{4} } \right)\omega_{2} }}\sin \left( {\theta_{1} } \right),$$

31 $$a_{2} \dot{\varphi }_{2} = \frac{{\alpha_{2} \varphi_{\rm I} a_{1} \omega_{1} }}{{2\left( {1 + G_{4} } \right)\omega_{2} }}\sin \left( {\theta_{1} } \right) - \frac{{\gamma_{3} a_{1} }}{{2\left( {1 + G_{4} } \right)\omega_{2} }}\cos \left( {\theta_{1} } \right) - \frac{{G_{4} \omega_{2} a_{2} }}{{2\left( {1 + G_{4} } \right)}}.$$

## Frequency response equations

An analysis is conducted on the frameworks $\dot{a}_{n} = 0\,,\,\dot{\theta }_{m} = 0\,(n = 1,2;m = 1,2)$. The frequency response equations that are displayed below were derived from the steady solutions and the periodic solution at the fixed points in Eqs. (28)–(31):

32 $$\begin{aligned} 0 = - & \left( {\frac{{\alpha_{1} a_{1} }}{{2\left( {1 + G_{2} } \right)}} + \frac{{\gamma_{1} \varphi_{\rm I} a_{1} }}{{2\left( {1 + G_{2} } \right)}} + \frac{{3G_{1} a_{1}^{3} \omega_{1}^{2} }}{{8\left( {1 + G_{2} } \right)}}} \right) + \frac{{\gamma_{1} a_{2} \omega_{2} }}{{2\left( {1 + G_{2} } \right)\omega_{1} }}\cos \left( {\theta_{1} } \right) - \frac{{\gamma_{2} a_{2} }}{{2\left( {1 + G_{2} } \right)\omega_{1} }}\sin \left( {\theta_{1} } \right) \\ - & \frac{f}{{2\left( {1 + G_{2} } \right)\omega_{1} }}\cos \left( {\theta_{2} } \right), \\ \end{aligned}$$

33 $$\begin{aligned} - \sigma_{2} a_{1} = - & \frac{{\gamma_{1} a_{2} \omega_{2} }}{{2\left( {1 + G_{2} } \right)\omega_{1} }}\sin \left( {\theta_{1} } \right) - \frac{{\gamma_{2} a_{2} }}{{2\left( {1 + G_{2} } \right)\omega_{1} }}\cos \left( {\theta_{1} } \right) - \frac{{G_{2} \omega_{1} a_{1} }}{{2\left( {1 + G_{2} } \right)}} + \frac{{\gamma_{2} \varphi_{\rm I} a_{1} }}{{2\left( {1 + G_{2} } \right)\omega_{1} }} \\ + & \frac{f}{{2\left( {1 + G_{2} } \right)\omega_{1} }}\sin \left( {\theta_{2} } \right), \\ \end{aligned}$$

34 $$0 = - \frac{{\alpha_{2} a_{2} }}{{2\left( {1 + G_{4} } \right)}} - \frac{{3G_{3} a_{2}^{3} \omega_{2}^{2} }}{{8\left( {1 + G_{4} } \right)}} + \frac{{\alpha_{2} \varphi_{\rm I} a_{1} \omega_{1} }}{{2\left( {1 + G_{4} } \right)\omega_{2} }}\cos \left( {\theta_{1} } \right) + \frac{{\gamma_{3} a_{1} }}{{2\left( {1 + G_{4} } \right)\omega_{2} }}\sin \left( {\theta_{1} } \right),$$

35 $$\left( {\sigma_{1} - \sigma_{2} } \right)a_{2} = \frac{{\alpha_{2} \varphi_{\rm I} a_{1} \omega_{1} }}{{2\left( {1 + G_{4} } \right)\omega_{2} }}\sin \left( {\theta_{1} } \right) - \frac{{\gamma_{3} a_{1} }}{{2\left( {1 + G_{4} } \right)\omega_{2} }}\cos \left( {\theta_{1} } \right) - \frac{{G_{4} \omega_{2} a_{2} }}{{2\left( {1 + G_{4} } \right)}}.$$

By solving the above nonlinear algebraic equations numerically with the FSOLVE code of MATLAB, we get the frequency response equations within the fixed solutions.

### Stability of system

To investigate the stability of the non-linear solution of the acquired fixed points.

36 $${\text{Let}}\;a_{n} = a_{n0} + a_{n1} (n = 1,2),\theta_{m} = \theta_{m0} + \theta_{m1} \left( {m = 1,2} \right),$$

where $a_{n0}$ and $\theta_{m0}$ are solutions of Eqs. (28)–(31), and $a_{n1}$, $\theta_{m1}$ are small perturbations, which are assumed to be small in comparison with $a_{n0}$ and $\theta_{m0}$.

Substituting Eq. (36) into Eqs. (28)–(31) with in just preserving the linear terms, the following equations and By using the trigonometric function and preserving only the linear terms $a_{10} ,a_{20} ,\theta_{10} ,\theta_{20}$ in Eqs. (32)–(35), we get the following system of first-order differential equations:

37 $$\begin{aligned} \dot{a}_{11} = - & \left( {\frac{{\alpha_{1} }}{{2\left( {1 + G_{2} } \right)}} + \frac{{\gamma_{1} \varphi_{\rm I} }}{{2\left( {1 + G_{2} } \right)}} + \frac{{9a_{10}^{2} G_{1} \omega_{1}^{2} }}{{8\left( {1 + G_{2} } \right)}}} \right)a_{11} + \left( { - \frac{{\gamma_{1} \omega_{2} a_{20} \sin \left( {\theta_{10} } \right)}}{{2\left( {1 + G_{2} } \right)\omega_{1} }} - \frac{{\gamma_{2} a_{20} \cos \left( {\theta_{10} } \right)}}{{2\left( {1 + G_{2} } \right)\omega_{1} }}} \right)\theta_{11} \\ + & \left( {\frac{{\gamma_{1} \omega_{2} \cos \left( {\theta_{10} } \right)}}{{2\left( {1 + G_{2} } \right)\omega_{1} }} - \frac{{\gamma_{2} \sin \left( {\theta_{10} } \right)}}{{2\left( {1 + G_{2} } \right)\omega_{1} }}} \right)a_{21} + \left( {\frac{f}{{2\left( {1 + G_{2} } \right)\omega_{1} }}\sin \left( {\theta_{20} } \right)} \right)\theta_{21} , \\ \end{aligned}$$

38 $$\begin{aligned} \dot{\theta }_{11} = & \;\left( {\frac{{\sigma_{2} }}{{a_{10} }} - \frac{{G_{2} \omega_{1} }}{{2a_{10} \left( {1 + G_{2} } \right)}} + \frac{{\gamma_{2} \varphi_{\rm I} }}{{2a_{10} \left( {1 + G_{2} } \right)\omega_{1} }} - \frac{{\alpha_{2} \varphi_{\rm I} \omega_{1} \sin \left( {\theta_{10} } \right)}}{{2a_{20} \left( {1 + G_{4} } \right)\omega_{2} }} + \frac{{\gamma_{3} \cos \left( {\theta_{10} } \right)}}{{2a_{20} \left( {1 + G_{4} } \right)\omega_{2} }}} \right)a_{11} \\ & + \left( { - \frac{{\gamma_{1} \omega_{2} a_{20} \cos \left( {\theta_{10} } \right)}}{{2a_{10} \left( {1 + G_{2} } \right)\omega_{1} }} + \frac{{\gamma_{2} a_{20} \sin \left( {\theta_{10} } \right)}}{{2a_{10} \left( {1 + G_{2} } \right)\omega_{1} }} - \frac{{\gamma_{3} a_{10} \sin \left( {\theta_{10} } \right)}}{{2a_{20} \left( {1 + G_{4} } \right)\omega_{2} }} - \frac{{\alpha_{2} \varphi_{\rm I} \omega_{1} a_{10} \cos \left( {\theta_{10} } \right)}}{{2a_{20} \left( {1 + G_{4} } \right)\omega_{2} }}} \right)\theta_{11} , \\ \end{aligned}$$

39 $$\begin{aligned} \dot{a}_{21} = & \left( {\frac{{\alpha_{2} \varphi_{\rm I} \omega_{1} \cos \left( {\theta_{10} } \right)}}{{2\left( {1 + G_{4} } \right)\omega_{2} }} + \frac{{\gamma_{3} \sin \left( {\theta_{10} } \right)}}{{2\left( {1 + G_{4} } \right)\omega_{2} }}} \right)a_{11} + \left( {\frac{{\gamma_{3} a_{10} \cos \left( {\theta_{10} } \right)}}{{2\left( {1 + G_{4} } \right)\omega_{2} }} - \frac{{\alpha_{2} \varphi_{\rm I} \omega_{1} a_{10} \sin \left( {\theta_{10} } \right)}}{{2\left( {1 + G_{4} } \right)\omega_{2} }}} \right)\theta_{11} \\ & - \left( {\frac{{\alpha_{2} }}{{2\left( {1 + G_{4} } \right)}} + \frac{{9G_{3} a_{20}^{3} \omega_{2}^{2} }}{{8\left( {1 + G_{4} } \right)}}} \right)a_{21} , \\ \end{aligned}$$

40 $$\begin{aligned} \dot{\theta }_{21} = & \left( {\frac{{\sigma_{2} }}{{a_{10} }} - \frac{{G_{2} \omega_{1} }}{{2a_{10} \left( {1 + G_{2} } \right)}} + \frac{{\gamma_{2} \varphi_{\rm I} }}{{2a_{10} \left( {1 + G_{2} } \right)\omega_{1} }}} \right)a_{11} + \left( { - \frac{{\gamma_{1} \omega_{2} a_{20} \cos \left( {\theta_{10} } \right)}}{{2a_{10} \left( {1 + G_{2} } \right)\omega_{1} }} + \frac{{\gamma_{2} a_{20} \sin \left( {\theta_{10} } \right)}}{{2a_{10} \left( {1 + G_{2} } \right)\omega_{1} }}} \right)\theta_{11} \\ & + \left( { - \frac{{\gamma_{1} \omega_{2} \sin \left( {\theta_{10} } \right)}}{{2a_{10} \left( {1 + G_{2} } \right)\omega_{1} }} - \frac{{\gamma_{2} \cos \left( {\theta_{10} } \right)}}{{2a_{10} \left( {1 + G_{2} } \right)\omega_{1} }}} \right)a_{21} + \left( {\frac{f}{{2a_{10} \left( {1 + G_{2} } \right)\omega_{1} }}\cos \left( {\theta_{20} } \right)} \right)\theta_{21} . \\ \end{aligned}$$

Equations (37) to (40) can be presented in the following matrix form:

41 $$\left[ {\begin{array}{*{20}c} {\dot{a}_{11} } & {\dot{\theta }_{11} } & {\dot{a}_{21} } & {\dot{\theta }_{21} } \\ \end{array} } \right]^{T} = \left[ J \right]\,\left[ {\begin{array}{*{20}c} {a_{11} } & {\theta_{11} } & {a_{21} } & {\theta_{21} } \\ \end{array} } \right]^{T} ,$$

42 $$\left[ J \right] = \left| {\begin{array}{*{20}c} {M_{1} } & {M_{2} } & {M_{3} } & {M_{4} } \\ {M_{5} } & {M_{6} } & 0 & 0 \\ {M_{7} } & {M_{8} } & {M_{9} } & 0 \\ {M_{10} } & {M_{11} } & {M_{12} } & {M_{13} } \\ \end{array} } \right|,$$

where $\left[ J \right]$ is the Jacobian matrix and $M_{n} \left( {n = 1,.....,13} \right)$ defined in Supplementary Appendix.

The stability of a given fixed point to a disturbance proportional to exp(λt) is determined by the roots of:

43 $$\left| {\begin{array}{*{20}c} {M_{1} - \lambda } & {M_{2} } & {M_{3} } & {M_{4} } \\ {M_{5} } & {M_{6} - \lambda } & 0 & 0 \\ {M_{7} } & {M_{8} } & {M_{9} - \lambda } & 0 \\ {M_{10} } & {M_{11} } & {M_{12} } & {M_{13} - \lambda } \\ \end{array} } \right| = 0.$$

Consequently, a non-trivial solution is stable if and only if the real parts of both eigenvalues of the coefficient matrix Eq. (41) are less than zero.

Which of the following polynomials has the following roots:

44 $$\lambda^{4} + \Gamma_{1} \,\lambda^{3} + \Gamma_{2} \,\lambda^{2} + \Gamma_{3} \lambda + \Gamma_{4} = 0,$$

where $\Gamma_{i} \left( {i = 1,....,4} \right)$ are the coefficients of Eq. (43). The Routh–Huriwitz criterion must be satisfied in order for the aforementioned system’s solution to be stable:

$$\Gamma_{1} > 0,\Gamma_{1} \Gamma_{2} - \Gamma_{3} > 0,\Gamma_{3} \left( {\Gamma_{1} \Gamma_{2} - \Gamma_{3} } \right) - \Gamma_{1}^{2} \Gamma_{4} > 0,\Gamma_{4} > 0.$$

## Results and discussion

In general vibration analysis is crucial in assessing the structural health and integrity of wind turbine towers. Here’s a technique commonly used for vibration analysis:

1. *Instrumentation placement*: Place accelerometers or vibration sensors at critical locations on the wind turbine tower. These locations typically include the base, mid-height, and top of the tower. The number and placement of sensors depend on the specific tower design and the level of detail required in the analysis.
2. *Data acquisition* Collect vibration data from the sensors over some time. This data can be gathered continuously or during specific operational conditions, such as during startup, normal operation, and shutdown.

But numerically we used different controls at worst case to determine the best control Systems to reduce vibration and protect the (WTT) from damage.

By applying the (RK-4) method and keeping a check on the system’s behavior these are the worst resonance cases $\left( {\omega_{1} = \omega_{2} } \right),\left( {\omega_{1} = \omega } \right)$ that is combination between Internal and Primary resonances, where the oscillations of the system and absorber have multi-limit cycles and increasing dynamic chaos. These are the conditions that must be controlled using the control to fade the vibration and pressure damage. This is demonstrated by the study of amplitude on the main system and choosing the worst cases without absorber. Because of this, the efficacy of several controls was compared, and the optimal one was selected in the event where the equation of motion, as shown in the accompanying figures.

**Figure 4:**
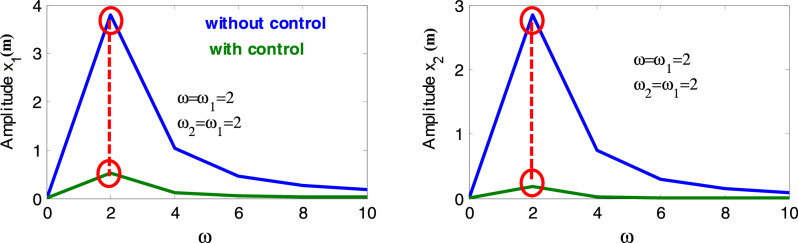
Diagram response to attain the measured one of worst resonance cases of the structure before and after the controller.

Figure 4 shows that the highest steady-state amplitude was achieved in one of the worst resonance cases before the inclusion of the controller. Nevertheless, after the incorporation of the CNVC and linear acceleration controller, the system amplitude appears to decrease when the controller operates in the presence of primary resonance and 1:1 internal resonance, which highlights the effectiveness of the CNVC and linear acceleration controller on the system.

**Figure 5:**
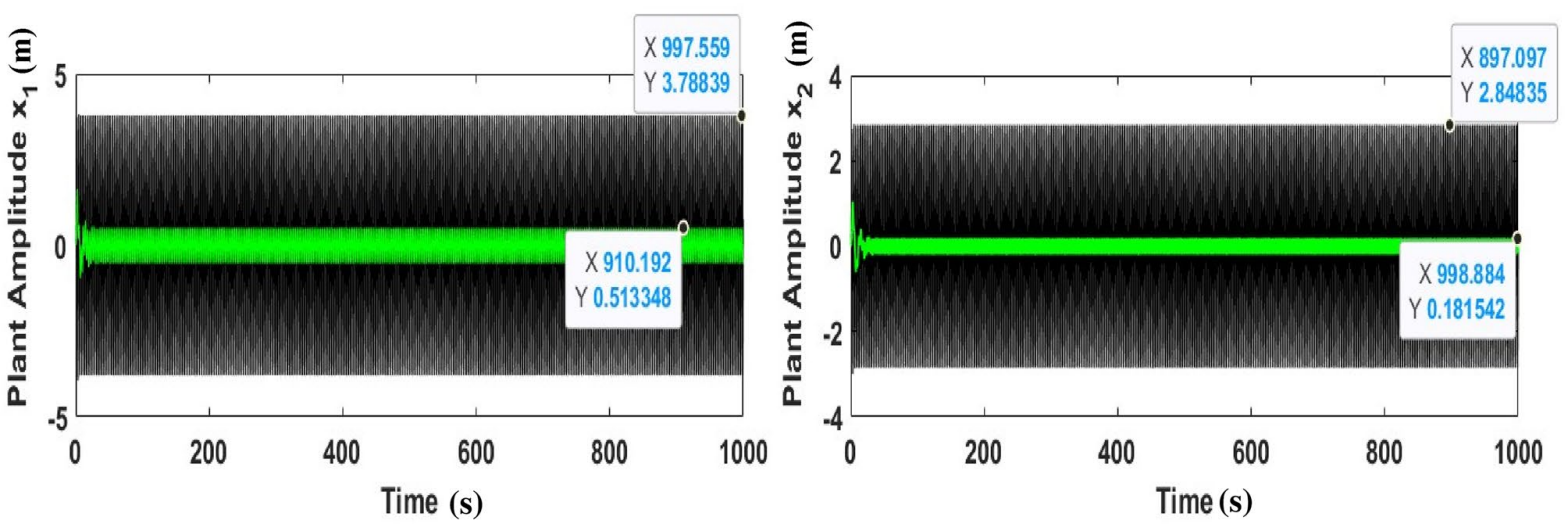
Effect of cubic-NVC and acceleration control on the system without control.

Figure 5 shows the chosen type of control is able to reduce the value of ($x_{1} = 3.788$ and $x_{2} = \, 2.84$) to approximately ($x_{1} = \, 0.51$ and $x_{2} = \, 0.18$), and according to the values of Table 1, it reduces ($x_{1} = 7.41$ and $x_{2} = 28.48$) times and ($x_{1}$ percentage 741% and $x_{2}$ percentage 2848%). This illustrates the effect of the chosen type of control (mix between Cubic NVC and Linear Negative Acceleration) and explains the extent of its control over the vibration amplitude. The important reasons that supported the choice of active control of wind turbines are that it is important to use a lightweight damper crossing, which is particularly important because adding a large amount to the top of the tower may lead to system instability and increase vibrations in the tower itself.

**Figure 6:**
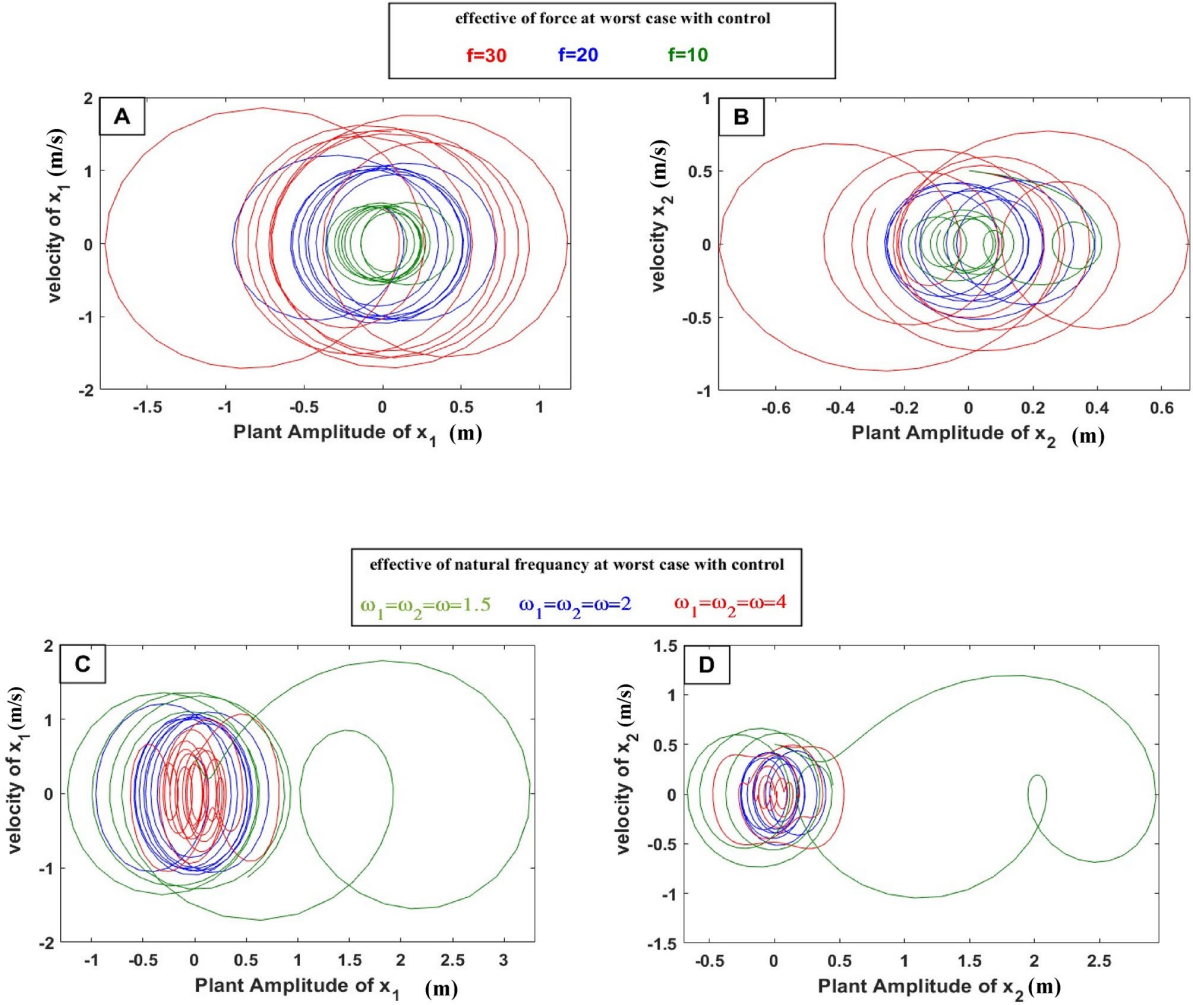
Effective of force and natural frequency on the phase plane.

In Fig. 6, the velocity and displacement phase plane of the time history of the controlled WTT system shown as multi-limit circles reach to ($\dot{x}_{1} =$ 1.2 and $\dot{x}_{2} =$ 0.22) m/s and ($x_{1} =$ 0.712 and $x_{2} =$ 0.39) m at the resonance region, respectively when, since the resonance amplification phenomenon is very serious near the natural frequency of WTT, we study the effect of force on Fig. 6A,B and the frequency resonance case at worst on Fig. 6C,D in phase plane, the result showed that by increasing force, the velocity and displacement resonance region will increase and become circles wider in addition to It becomes more vulnerable to vibrations and dynamic disorder, and it’s hard to control the stability of the system. By decreasing ɷ the velocity and displacement resonance region will increase and become circles wider and fewer. That’s the goal of reducing the speed and force of vibration to reduce the risk of system destruction.

**Figure 7:**
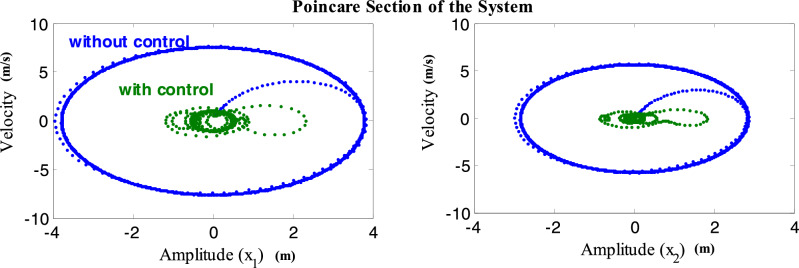
Poincare map of style without and with controller at *ω* = *ω*_1_ = *ω*_2_.

Figure 7 depicts the phase portrait, which illustrates the relationship between velocity and amplitude, as well as a Poincaré map diagram, for the system before and after applying the CNVC and LNAC. This figure displays the chaotic attractor and estimates multi-limit cycle. In addition, the reaction of the WTT model to the CNVC and LNAC is depicted in the Poincaré map shown in Fig. 7, which illustrates the type of motion for the model and the control.

### Frequency response curve

**Figure 8:**
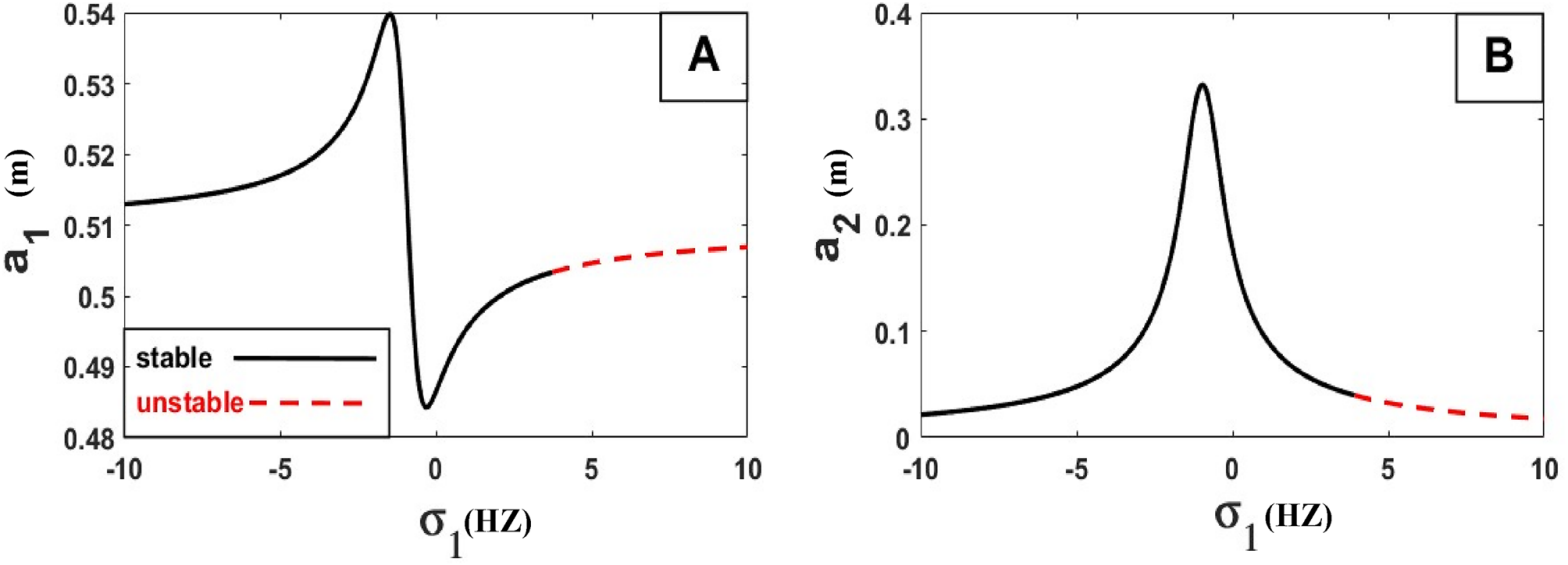
Frequency response curves.

**Figure 9:**
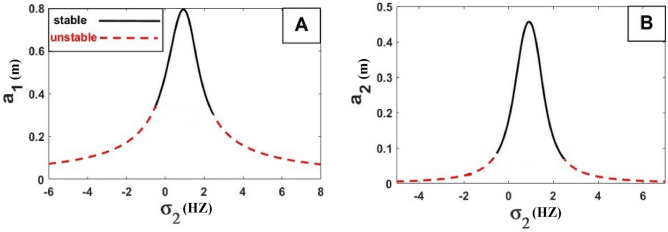
Frequency response curves.

**Figure 10:**
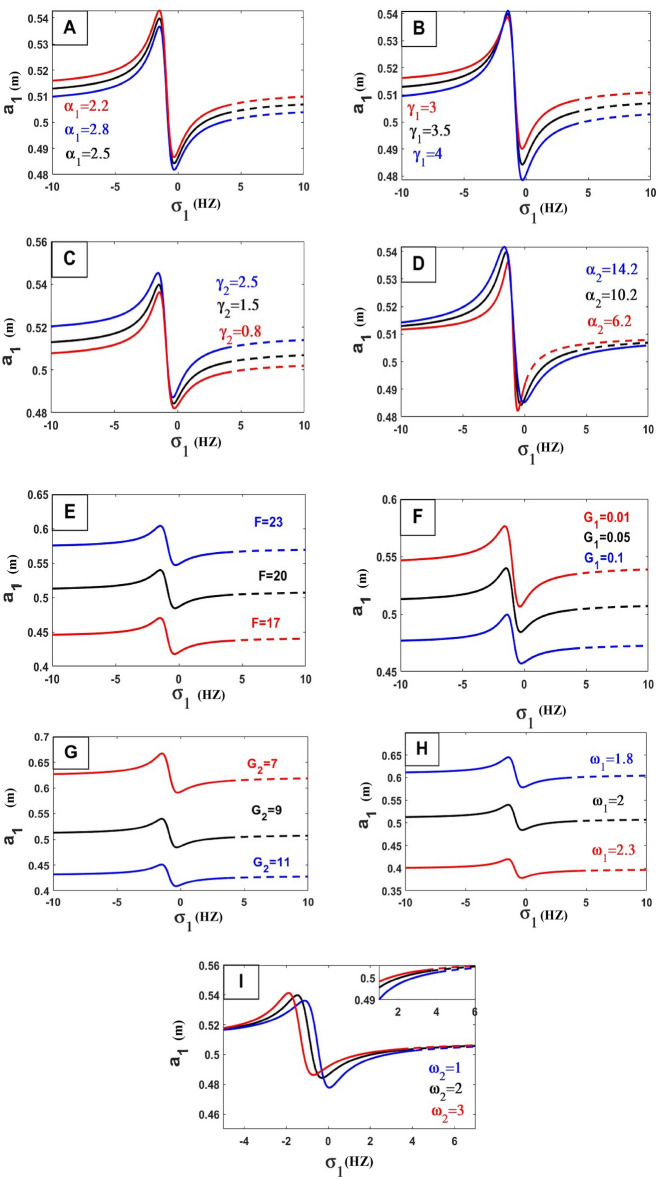
Effects of parameters on the response curves of stability of the steady-state amplitude $a_{1}$ against $\sigma_{1}$.

**Figure 11:**
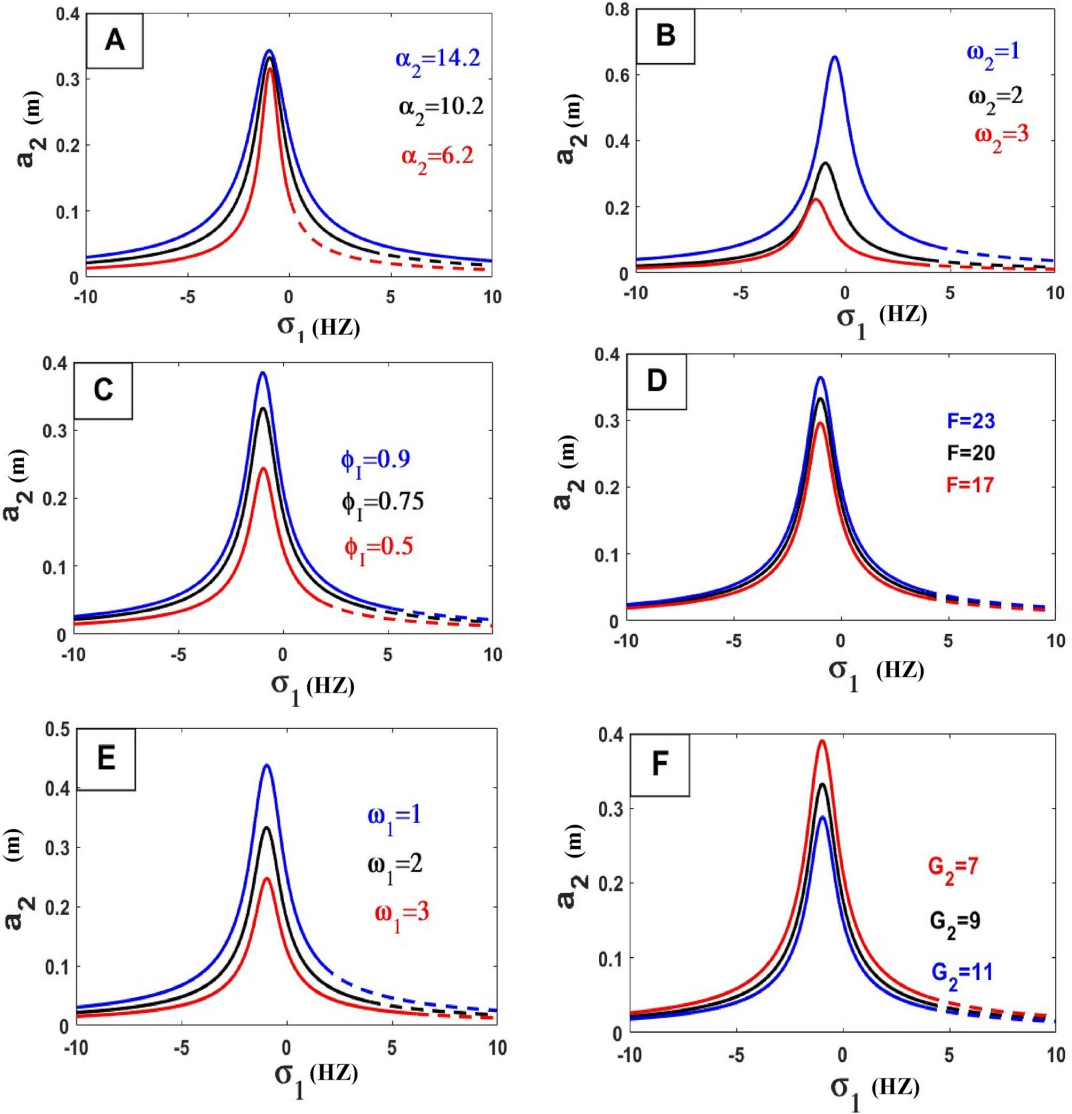
Effects of parameters on the response curves of stability of the steady-state amplitude $a_{2}$ against $\sigma_{1}$.

**Figure 12:**
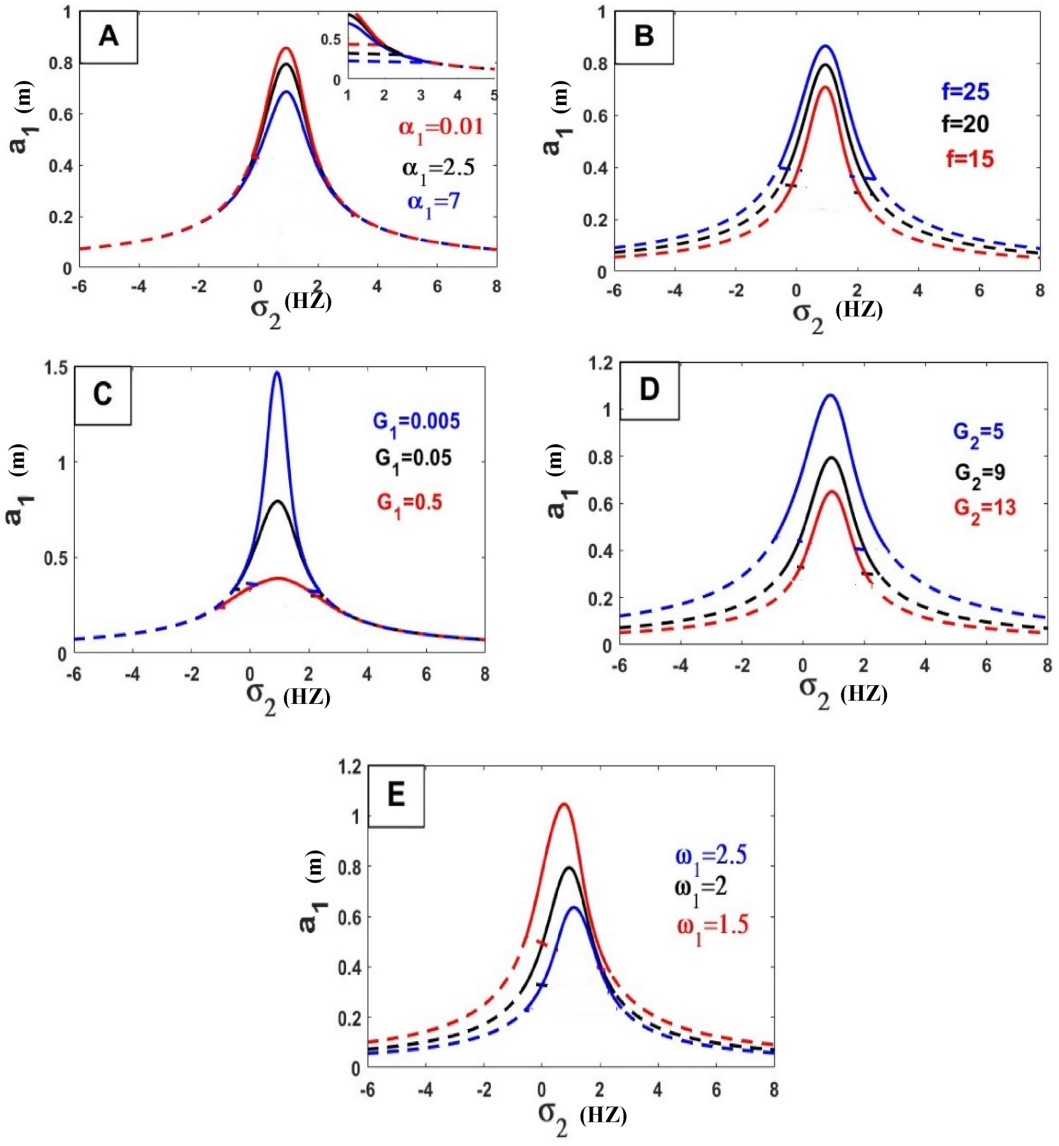
Effects of parameters on the response curves of stability of the steady-state amplitude $a_{1}$ against $\sigma_{2}$.

**Figure 13:**
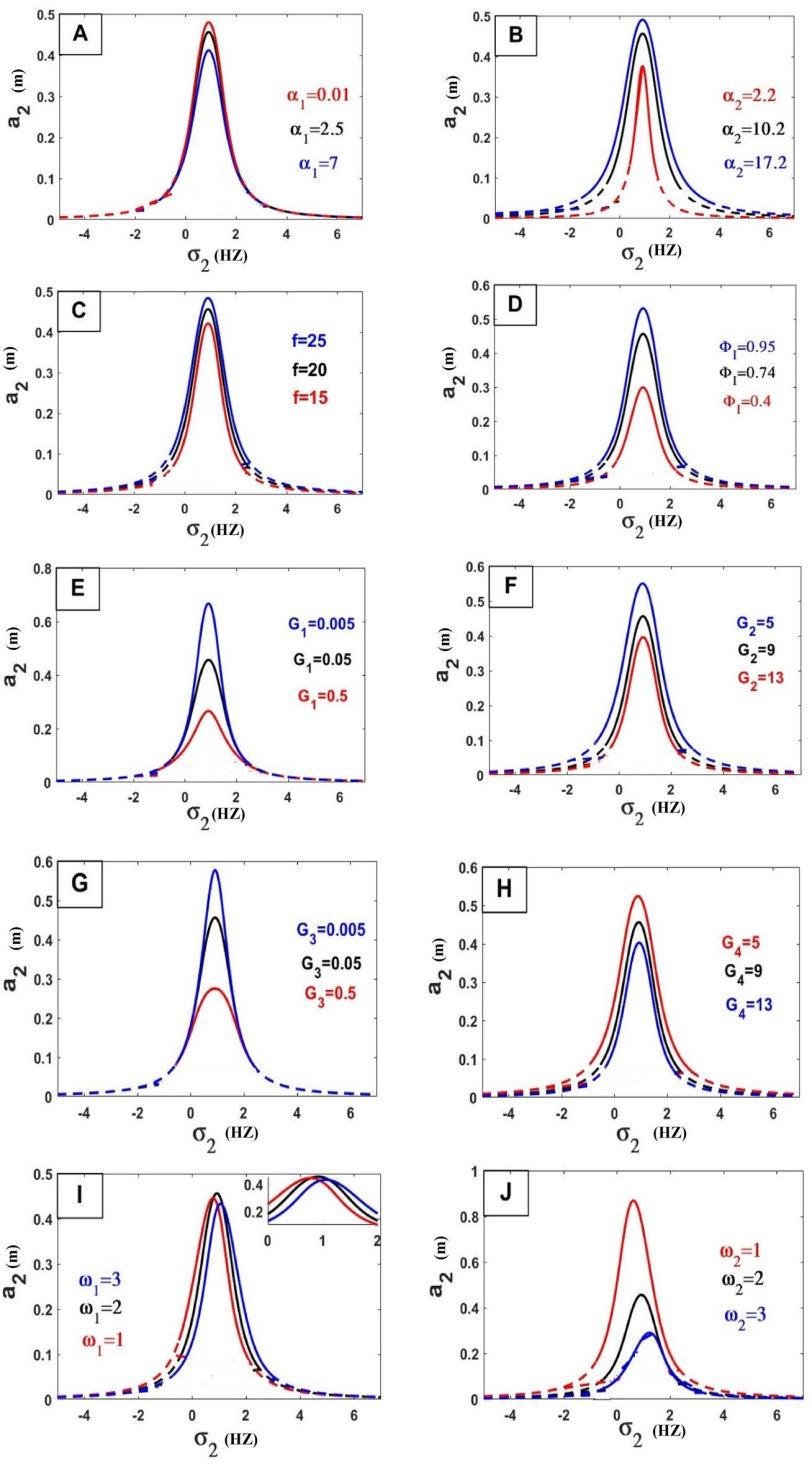
Effects of parameters on the response curves of stability of the steady-state amplitude $a_{2}$ against $\sigma_{2}$.

The frequency equation is represented graphically by using numerical methods. The frequency response equation is a nonlinear algebraic equation that is solved numerically by using the Newton-Rapson method. The frequency response Eqs. (32)–(35) are nonlinear algebraic equations, the results are shown in Figs. 8, 9, 10, 11, 12 and 13 for the steady-state amplitudes of the frequency response curve $a_{1} ,a_{2}$ against parameter $\sigma_{1}$ and the frequency response curve $a_{1} ,a_{2}$ against parameter $\sigma_{2}$ resonance $\left( {\omega_{1} = \omega_{2} } \right),\left( {\omega_{1} = \omega } \right)$. The study of the changes in parameter values shows that the period of stability and instability changes with the different values of parameters the same goes for frequency values.

Figure 8 illustrates the relationship between amplitude and sigma (A) stability of the steady-state amplitude ($a_{1}$ against $\sigma_{1}$), ($a_{2}$ against $\sigma_{1}$) in Figure (B) demonstrating how the periods of stability and instability changes and how the sable at the all of the curves except the rightmost part of the curve unstable.

Figure 9 illustrates the relationship between amplitude and sigma (A) stability of the steady-state amplitude ($a_{1}$ against $\sigma_{2}$), ($a_{2}$ against $\sigma_{2}$) in Figure (B) demonstrating how the periods of stability and instability change and how the sable at the peak of the curve is at the top of the left and right branches and The lower part of the left and right branches is unstable.

### Effect of different parameters

In the following Figs. 10, 11, 12 and 13, our investigation focused on examining the extent to which variables affect the frequency curve and its impact on the areas of stability and instability and we have concluded the following:

1. *Natural frequency* $\left( {\omega_{n}^{2} = \frac{{k_{n} }}{{m_{n} }}} \right)$ all bodies have natural frequencies because all bodies have mass and stiffness’s. Mechanical vibration is essentially a play between inertial and elastic forces. Physically the amplitude of the wave is inversely proportional to its frequency because of the interaction between the two. As the frequency increases the amplitude decreases. As the frequency drops, the amplitude rises. The oscillation’s amplitude is independent of its frequency. This was confirmed by the results of the study of the effect of natural frequency (ɷ_1_, ɷ_2_) in Fig. 10H,I) relation (amplitude $a_{1}$ against $\sigma_{1}$) and in Fig. 11B,E) in relation (amplitude $a_{2}$ against $\sigma_{1}$) by increasing these values, the amplitude-frequency decreases. And that’s also what happens in relation (amplitude $a_{1}$ against $\sigma_{2}$) and in Fig. 12E in ɷ_1_ and in Fig. 13I,J relation (amplitude $a_{2}$ against $\sigma_{2}$) by increasing these values, the amplitude-frequency decreases.
2. In physics, *damping* is the process of releasing energy to stop vibratory motion, including noise, alternating electric currents, and mechanical oscillations. The amplitude gradually decreases by increasing values of damping because of the increased damping force within the elastic limit of mainframes, unless there is damage to the system controlled by the vibration. This was confirmed by the results of the study of the effect of damping coefficient ($\alpha_{1}$, $\alpha_{2}$) in Fig. 10A,D relation (amplitude $a_{1}$ against $\sigma_{1}$) and in Fig. 11A in relation (amplitude $a_{2}$ against $\sigma_{1}$) by increasing these values, the amplitude-frequency decreases with $\alpha_{1}$ and in $\alpha_{2}$ increasing amplitude with increase $\alpha_{2}$. And that’s also what happens in relation (amplitude $a_{1}$ against $\sigma_{2}$) and in Fig. 12A in relation (amplitude $a_{1}$ against $\sigma_{2}$) in $\alpha_{1}$ decreases amplitude with increasing $\alpha_{1}$ and in Fig. 13A,B relation (amplitude $a_{2}$ against $\sigma_{2}$) by increasing values of $\alpha_{1}$, the amplitude-frequency decreases and opposite occurs with $\alpha_{2}$.
3. *The stiffness constant* is the ratio of the center of mass displacement to the restoring force given to a basic harmonic oscillator system. The amount of resistance an item has to deformation when a force is applied is known as its stiffness. An axial force causes a coil spring to extend. The complementary idea is flexibility or pliability: the more flexible an object is, the less stiff it is. So that by increasing the amplitude increased This was confirmed by the results of the study of the effect of stiffness ($\gamma_{1} ,\gamma_{2}$) in Fig. 10B,C relation (amplitude $a_{1}$ against $\sigma_{1}$).
4. A force is described in *physics* as an effect that acts on an item and modifies its state, direction, location, or movement. An object with mass can modify its velocity, or accelerate, in response to a force. According to this definition, the relationship between force and amplitude is Expulsion Relationship by increasing force values increases amplitude values, This was confirmed by the results of the study of the effect of coefficient of force $f$ in Fig. 10E relation (amplitude $a_{1}$ against $\sigma_{1}$), in Fig. 11D in relation (amplitude $a_{2}$ against $\sigma_{1}$), in Fig. 12B in relation (amplitude $a_{1}$ against $\sigma_{2}$) and in Fig. 13C relation (amplitude $a_{2}$ against $\sigma_{2}$).
5. *Gain of control* (Active control), generally control used to reduce vibration and damage to the dynamical system, this shows that the relationship is inversely formed by increasing the values of the gain of control by decreasing amplitude, This was confirmed by the results of the study of the effect of the control coefficients ($G_{1} ,G_{2} ,G_{3} ,G_{4}$) in Fig. 10F,G relation (amplitude $a_{2}$ against $\sigma_{1}$), in Fig. 11F on relation (amplitude $a_{2}$ against $\sigma_{1}$), in Fig. 12C,D in relation (amplitude $a_{1}$ against $\sigma_{2}$) and Fig. 13E–H relation (amplitude $a_{2}$ against $\sigma_{2}$).
6. $\Phi_{I}$ vibration mode at the position of IP-TMD, effect on the amplitude very clearly by increasing values of this parameter the amplitude increased, This was confirmed by the results in Fig. 11C in relation (amplitude $a_{2}$ against $\sigma_{1}$) and Fig. 13D (amplitude $a_{2}$ against $\sigma_{2}$).

**Figure 14:**
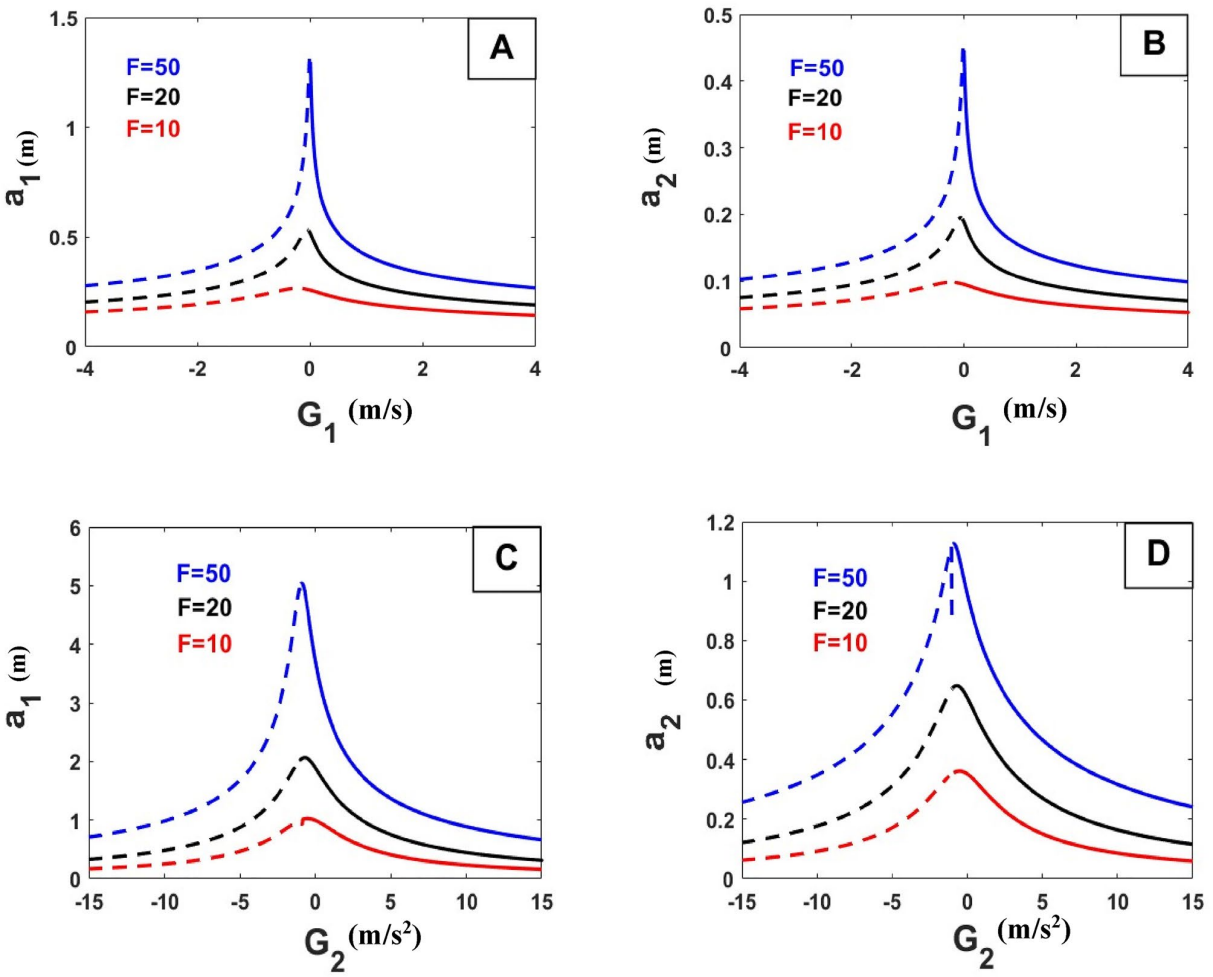
Effect of force on the relation between the amplitude and parameters of control.

Figure 14 shows the extent of the effect of the control gain on the amplitude in different cases of force values, whatever the power affects the system, and selected control values can keep the body in a stable state.

## Comparison

In this section, we conducted various types of comparisons, to demonstrate the validity of our studies.

### Comparison of types of control

**Figure 15:**
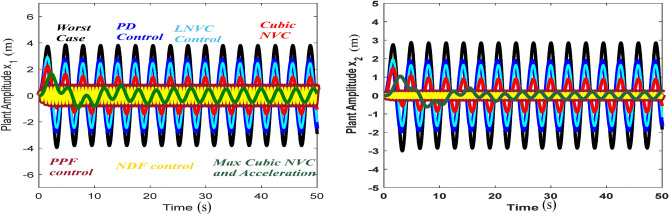
Effects of different types of control on the system without control.

Figure 15 shows the outcome resulting from the types of control methods applied to the worst-case situation. To enable us to select the control that has the most impact on the system, different types are compared. We examine PPF, or Cubic NVC, NDF, PD, LNVC, and LNAC as seen in the above representation. Combining LNAC with CNVC proved to be the best control which successful approach to controlling and reducing vibration, as shown by the results.

### Comparison between the numerical and approximation solutions

**Figure 16:**
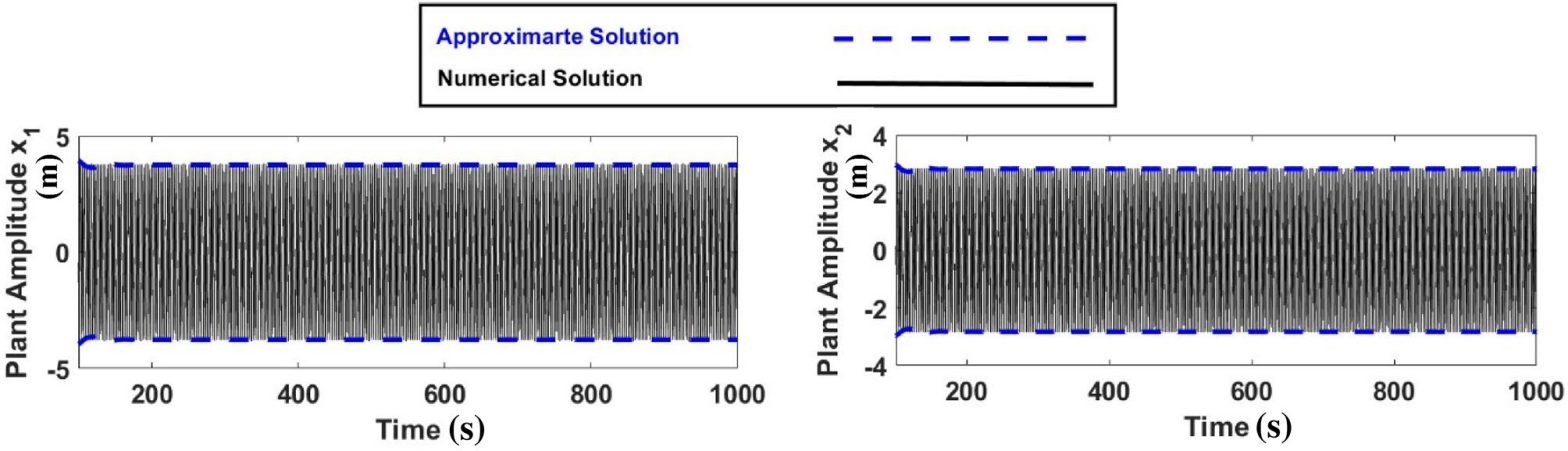
Comparison between the numerical and approximation solutions at the worst case without control.

Figure 16 shows that by comparing the numerical solution with the approximation solution in the worst case without and with control, we were able to reach good integrating conclusions and practically identical results.

### Comparison between frequency response curves (FRC) and time history using RK-4

**Figure 17:**
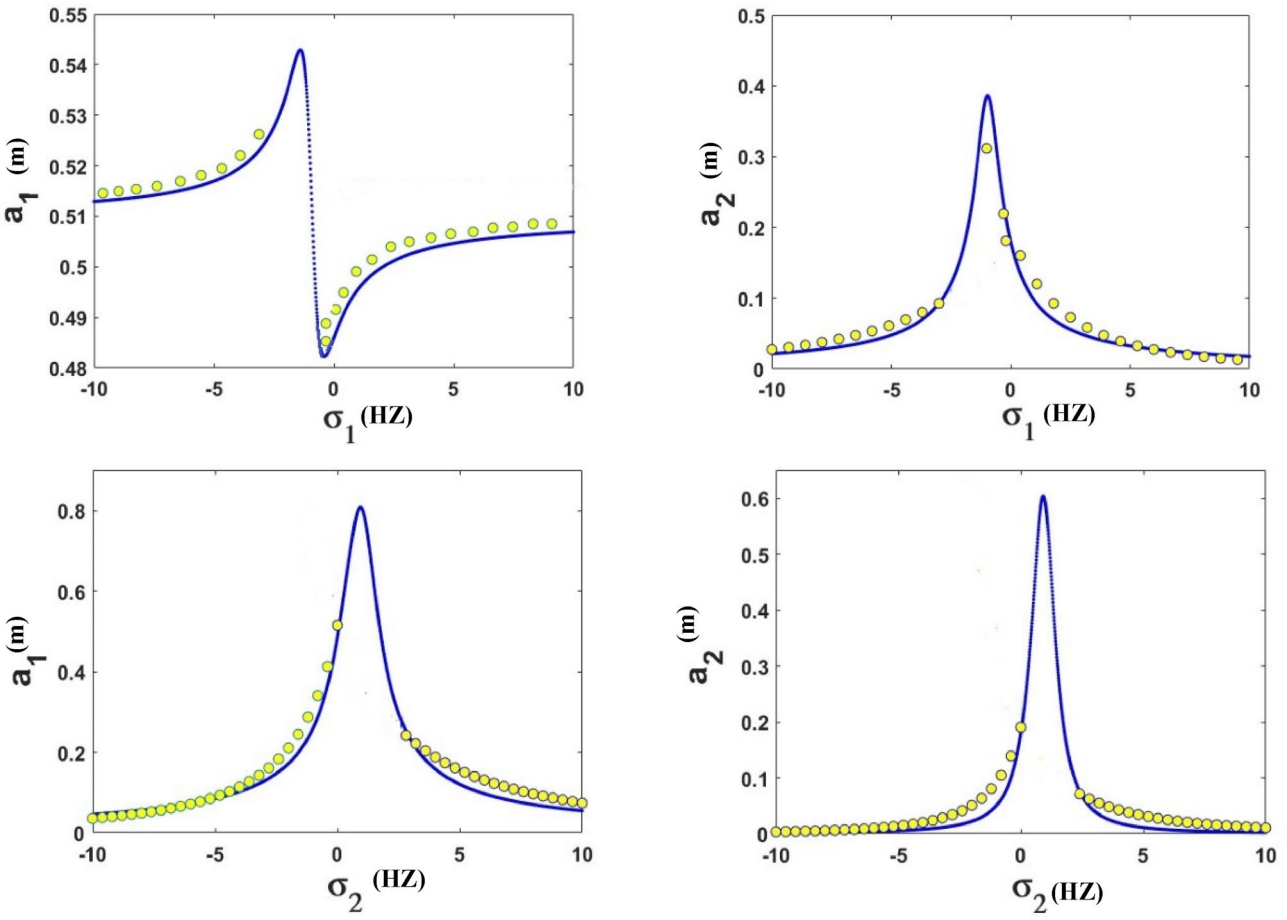
Comparison of frequency response curve FRC (blue line) and numerical solution using RK-4 (yellow circle).

Figure 17 confirms the results of Figs. 8 and 9 numerically. It is demonstrated that the numerical simulation results match well with the analytical solution results.

**Figure 18:**
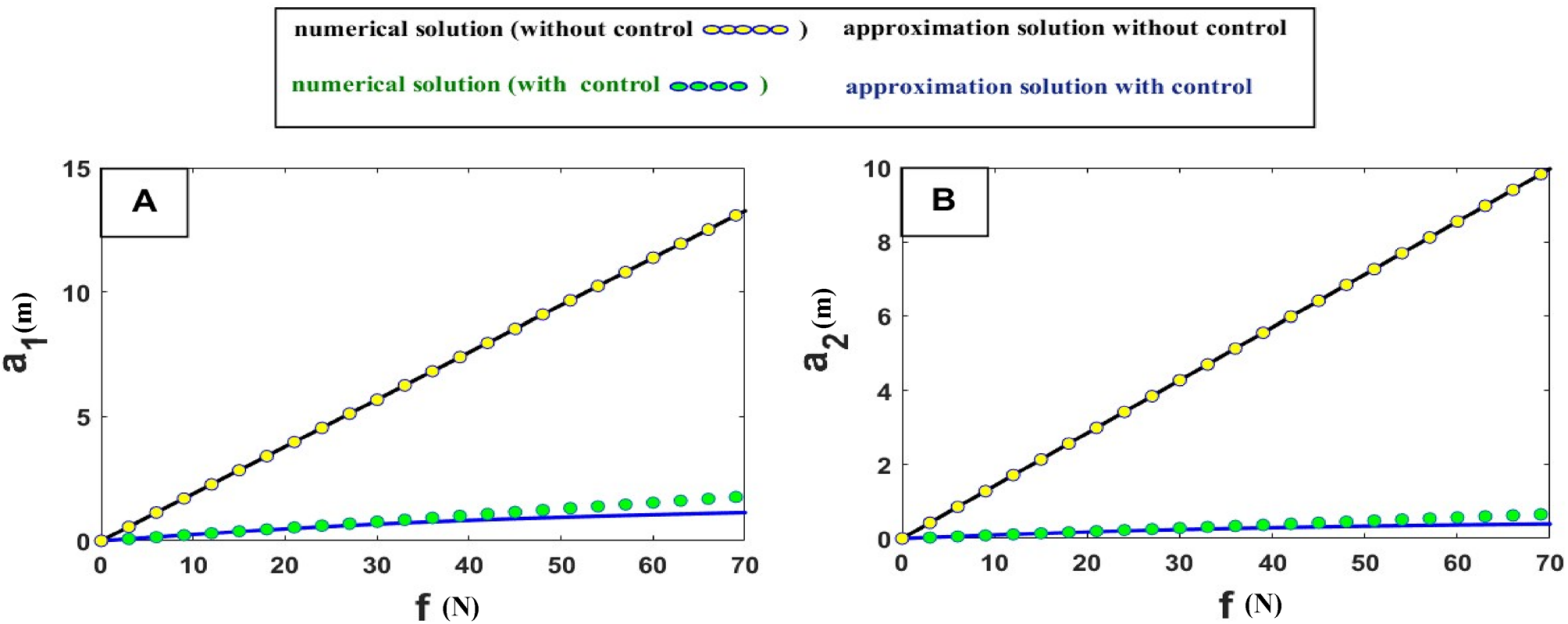
Effect of force on the amplitude.

Figure 18 study’s the relationship between (amplitude $\left( {a_{1} ,a_{2} } \right)$ and force), an addition to, firstly: Comparison between the numerical solution and approximation solution before and after adding control, the comparison showed that The analytical solution result and the numerical simulation result complement accurately. Secondly: Studying the relationship between amplitude and force before and after adding control demonstrated that after control was added, where force was regulated in such a way that the system was stable before control, with an increase in force by increasing the amplitude value (as shown when force was equal to 70 up to the $a_{1}$ value of 12 and $a_{2}$ value of 10), but after the addition of control, no matter how much force increased, the amplitude remained low (as shown when force was equal to 70 up to the $a_{1}$ value of 0.9 and $a_{2}$ value of 0.5).

## Conclusions

The study’s approximated methodology (averaging method) generated an approximate response when the response equation was shown. A numerical examination of the system’s behavior was part of the research to determine whether or not the controller exists.

The phase plane methodology and the frequency response equation of the (averaging method) are employed concurrently with the resonance case study to evaluate the numerical solution and show the stability of the system. Based on the above study’s present implementation, which has an impact on the frequency response equations and findings, the following conclusions are made:

1. As can be seen in the following illustration, the most effective control method for regulating and minimizing vibration, which combined LNAC with CNVC vibration control.
2. We obtained solid integrating findings and almost equal results by comparing the numerical solution with the approximation solution in the worst case without control.
3. Confirms the results of Figs. 8 and 9 numerically, it is demonstrated that, in two relations (A) relation between ($a_{1}$ and $a_{2}$ with $\sigma_{1}$) and (B) relation between ($a_{1}$ and $a_{2}$ with $\sigma_{2}$), the numerical simulation results match well with the analytical solution result.
4. Tables 2 and 3 confirm the comparison of the obtained results between FRC solution and RK-4 solution
5. The graphical representation of the frequency equation is achieved through numerical methods. By examining the impact of parameter values, it is clear that the period of stability and instability changes with varying parameter values, as does the frequency values.
6. The study of relationship between amplitude and force results showed that the analytical solution and numerical simulation effectively complemented the control method, providing accurate and reliable outcomes.
7. The phase plane of the time history of the controlled WTT system shown as multi-limit circles since the resonance amplification phenomenon is very serious near the natural frequency of WTT.

**Table 2:** Comparison of the obtained results between FRC solution and RK-4 solution for $a_{1}$.

$\sigma_{2}$	RK-4 solution	FRC solution	Absolute error
− 5	0.0849010921152189	0.32615432	0.24125323
− 4.7	0.0894428818656473	0.40865891	0.31921603
− 4.4	0.0944960209647243	0.53730456	0.44280854
− 4.1	0.100151576612580	0.73121638	0.6310648
− 3.8	0.106523467010785	0.55439994	0.44787648
− 3.5	0.113756049422030	0.67822087	0.56446482
− 3.2	0.122034913772403	0.66328363	0.54124871
− 2.9	0.131602557073843	0.52089936	0.3892968
− 2.6	0.142781672607980	0.62746653	0.48468486
2.3	0.156010636654427	0.93725286	0.78124222
3	0.0849010921152189	0.32615432	0.24125323
4.4	0.0894428818656473	0.40865891	0.31921603
4.7	0.0944960209647243	0.53730456	0.44280854
4.8	0.100151576612580	0.73121638	0.6310648
5	0.106523467010785	0.55439994	0.44787648

**Table 3:** Comparison of the obtained results between FRC solution and RK-4 solution for $a_{2}$.

$\sigma_{2}$	RK-4 solution	FRC solution	Absolute error
− 5	0.00549459203449567	0.09739295	0.09189835
− 4.7	0.00609613872569644	0.13731265	0.13121651
− 4.4	0.00680184765406233	0.20289669	0.19609484
− 4.1	0.00763714318516146	0.31741515	0.30977801
− 3.5	0.00984270715528906	0.0168229	0.00698019
− 3.2	0.0113202686266494	0.30165695	0.29033668
− 2.6	0.0154715106897420	0.46202416	0.44655265
2.5	0.0677196605127895	0.02102133	0.046698328
2.8	0.0493036042683970	0.01741454	0.03188906
3.1	0.0373951655855542	0.01457652	0.022818643
3.4	0.0292898754525218	0.01232468	0.016965194
3.7	0.0235395049242276	0.01050458	0.013034924
4	0.0193193300857584	0.00904163	0.010277699
4.3	0.0161338906186519	0.00783144	0.008302454
4.6	0.0136723253347886	0.00682152	0.006850808
4.9	0.0117317289078956	0.00598895	0.005742781

### Supplementary Information

Supplementary Information.

## Data Availability

All data generated or analyzed during this study are included in this manuscript.

## List of symbols

$K_{1}$: Stiffness of system
$K_{2}$: The stiffness coefficient of springs
$C_{1}$: Damping of system
$C_{2}$: The damping coefficient of viscous dampers
$x_{1} ,x_{2}$: Displacements of WTT system and inner platform (IP)-tuned mass damper (TMD)
$\ddot{x}_{1} ,\ddot{x}_{2}$: The acceleration of $x_{1} ,x_{2}$
$$\omega_{1} ,\omega_{2}$$: Natural frequencies
$\omega$: The external excitation frequency
$G_{1} ,G_{3}$: Non-linear gains of the cubic negative velocity controller
$G_{2} ,G_{4}$: Linear gains of the negative acceleration controller
$m_{1} ,m_{2}$: The generalized mass and physical mass of IP-TMD
$F_{eff}$: The external excitation force $F_{eff} \left( t \right) \, = \, P_{0} \,{\text{sin}}\left( {\omega t} \right)$
$P_{0}$: The excitation amplitude
$\varphi_{I}$: The shape value of the first vibration mode at the position of IP-TMD
$V_{A1}$: Approximate solution before control
$V_{A2}$: Approximate solution after control
